# Hyperpolarizabilities of Push–Pull Chromophores in Solution: Interplay between Electronic and Vibrational Contributions †

**DOI:** 10.3390/molecules27248738

**Published:** 2022-12-09

**Authors:** Tomáš Hrivnák, Miroslav Medveď, Wojciech Bartkowiak, Robert Zaleśny

**Affiliations:** 1 Department of Molecular Simulations of Polymers, Polymer Institute, Slovak Academy of Sciences, SK-845 41 Bratislava, Slovakia; 2 Department of Chemistry, Faculty of Natural Sciences, Matej Bel University, SK-974 00 Banská Bystrica, Slovakia; 3 Regional Centre of Advanced Technologies and Materials, Faculty of Science, Palacký University Olomouc, 783 71 Olomouc, Czech Republic; 4 Department of Physical and Quantum Chemistry, Faculty of Chemistry, Wrocław University of Science and Technology, Wyb. Wyspiańskiego 27, PL-50370 Wrocław, Poland; 5 Faculty of Chemistry, Wrocław University of Science and Technology, Wyb. Wyspiańskiego 27, PL-50370 Wrocław, Poland

**Keywords:** hyperpolarizability, solvent effects, vibrational contributions, anharmonicity, push–pull chromophores, density functional theory

## Abstract

Contemporary design of new organic non-linear optical (NLO) materials relies to a large extent on the understanding of molecular and electronic structure–property relationships revealed during the years by available computational approaches. The progress in theory—hand-in-hand with experiment—has enabled us to identify and analyze various physical aspects affecting the NLO responses, such as the environmental effects, molecular vibrations, frequency dispersion, and system dynamics. Although it is nowadays possible to reliably address these effects separately, the studies analyzing their mutual interplay are still very limited. Here, we employ density functional theory (DFT) methods in combination with an implicit solvent model to examine the solvent effects on the electronic and harmonic as well as anharmonic vibrational contributions to the static first hyperpolarizability of a series of push–pull α,ω-diphenylpolyene oligomers, which were experimentally shown to exhibit notable second-order NLO responses. We demonstrate that the magnitudes of both vibrational and electronic contributions being comparable in the gas phase significantly increase in solvents, and the enhancement can be, in some cases, as large as three- or even four-fold. The electrical and mechanical anharmonic contributions are not negligible but cancel each other out to a large extent. The computed dynamic solute NLO properties of the studied systems are shown to be in a fair agreement with those derived from experimentally measured electric-field-induced second-harmonic generation (EFISHG) signals. Our results substantiate the necessity to consider concomitantly both solvation and vibrational effects in modeling static NLO properties of solvated systems.

## 1. Introduction

During the past decades, organic nonlinear optical (NLO) chromophores have become a full-valued alternative to their inorganic counterparts owing to several attractive features, such as low dielectric constants, weak frequency dispersion of their refractive indices and higher-order susceptibilities, and extremely fast response times. These features are mainly related to a dominant electronic contribution to optical (hyper)polarizability of these systems, unlike the inorganic NLO materials with the response usually arising from lattice vibrations. In addition, a high versatility of organic compounds enables fine tuning of properties and designing structures for specific purposes, including the integration into various hybrid materials such as (bio)polymers, Langmuir–Blodgett films (LBFs) [1], self-assembled monolayers (SAMs) [2,3], and metal–organic frameworks (MOFs) [4,5]. Continuous joint experimental and theoretical efforts to rationalize structure–property relationships have led to successful design of new organic materials with the NLO figures of merit (FOM) significantly higher than those of the inorganic systems [6,7]. To further increase FOM and thus to enhance the application potential of newly designed NLO materials, the molecular modeling must include all relevant physical aspects of the studied NLO processes and describe them at an appropriate level of accuracy. For instance, it is nowadays well established that the vibrational contributions to frequency-independent (static) hyperpolarizabilities can be as large as or even larger than their electronic counterparts [8,9], and the anharmonic effects might be significant even for weakly anharmonic systems [10], especially if the static or low-frequency dependent (e.g., in the IR region) electric fields are present in the NLO process of interest [8,11]. Given the scope of the present study, it should be underlined that these findings have been mostly acquired by analyzing the gas-phase NLO properties.

On the other hand, there is a strong experimental and computational evidence that the solute–solvent interactions can dramatically modify the electronic NLO properties of the solute molecules [12,13,14,15,16,17,18,19,20]. To account for the solvation effects, the computational modeling relies on either implicit or explicit solvation models (or their hybrids) [21,22,23,24,25,26,27,28,29,30]. In the former, the solute molecule occupies a cavity created in a homogeneous medium representing the solvent, and the solute–solvent interactions are reduced to a mutual interplay between the solute electron density described at the quantum mechanical (QM) level and the cavity surface charges, which makes the implicit models computationally highly efficient. A drawback of these models is that the simplified description of the solute–solvent interface can capture neither specific intermolecular forces (e.g., hydrogen bonds) nor possible structural peculiarities of the solvation shell [17]. Apart from such cases, the implicit solvation models were proved to be a highly powerful tool also in the field of NLO properties, with the polarizable continuum model (PCM) [19,23,24] at the forefront of popularity. It should be underlined that these studies largely focus on the electronic (hyper)polarizabilities, while the inclusion of vibrational contributions for solvated NLO chromophores is far from being routine [15,31,32], not only because of significantly higher computational demands but also due to the fact that the appropriate solvation regimes should be assumed for static and dynamic responses, as shown by Egidi et al. for molecular polarizabilities [33]. In the explicit solvation models, a solute molecule is surrounded by a sufficiently large number of solvent molecules, and the NLO responses are evaluated in a sequential manner involving molecular dynamics (MD) or Monte Carlo (MC) simulations followed by the computation of required properties for a representative set of snapshots [27,28,29,30,32,34,35]. Within this framework, the vibrational effects might be partially included via the dynamics but can also be evaluated explicitly [32]. In any case, such a strategy is computationally rather demanding, and therefore usually recommended only if the implicit solvation models are inadequate.

**Figure 1:**
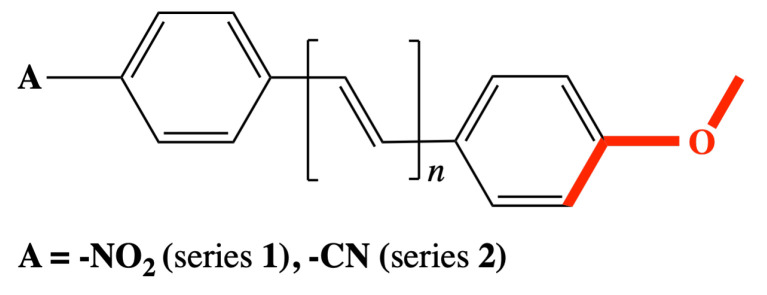
Molecules studied in the present work and their labeling. Dihedral angle (in red) is 180 and 0 deg for conformers **I** and **II**, respectively.

As outlined above, the technology-motivated interests in NLO organic materials resulted in numerous experimental and theoretical studies aiming at analyses of environmental effects on molecular nonlinear responses to electromagnetic perturbations. In particular, we have learned that electronic contributions to (hyper)polarizabilities of π-conjugated organic molecules might be fine tuned by solvent polarity [36,37]. Despite these efforts and theory developments, the solvents effects on vibrational contributions to (hyper)polarizabilities were scarcely studied [38], which is particularly true in the case of anharmonic corrections [33]. The present study aims at filling this gap and presents the results of investigations of the solvent effects on the vibrational NLO properties of organic molecules, including the anharmonic contributions. To that end, we chose a series of α,ω-diphenylpolyene oligomers (Figure 1) containing a methoxy group and nitro (series **1**)/cyano (series **2**) substituents as electron donor and electron acceptor groups, respectively, as representative push–pull NLO systems, which were experimentally studied by Cheng et al. [39,40]. The design of NLO chromophores based on linking the electron donating and electron withdrawing moieties by a π-conjugated chain is a common strategy ensuring the fundamental requirements for high electronic second-order NLO responses, i.e., the efficient electron delocalization and the molecular asymmetry [13,41,42,43,44,45,46,47]. The shortest studied oligomers belong to a large family of stilbenes, featuring a unique coupling of two aromatic moieties via a carbon–carbon double bond. Due to their high versatility enabling to tune the electronic and optical properties of the material, stilbenes and their derivatives have found numerous practical applications in the dye industry, paper and textile processing, and optoelectronic devices, but also as pharmaceutics and antitumor agents, molecular probes and labels for proteins and biomembranes [48]. It is worth noting that stilbenes have also been intensively investigated due to their optical [49] and, in particular, photochromic properties [50,51,52,53,54,55,56,57,58]. The *E/Z*-photoisomerization of stilbene is a prototypical unimolecular reaction for the studies of reaction dynamics both in the gas and condensed phases. In the case of push–pull derivatives, the quantum yields of the E→Z photoisomerization decrease with the solvent polarity, which was attributed to a concurrent twisted intramolecular charge transfer (TICT) relaxation pathway [55]. On the other hand, the reverse Z→E photoisomerization competes with a 6π-electrocyclization leading to dihydrophenanthrene [58], which readily oxidizes to phenanthrene. To avoid the undesired processes lowering the quantum yields, stiff-stilbenes, i.e., sterically restricted fused ring analogs of stilbene were proposed as more suitable for photoswitching applications [59,60,61]. By incorporating donor–acceptor substituents, their operational region was shifted to the visible light, and the reversible protonation of the donor unit allowed gating of this photoswitching process [62]. The attractive photochromic properties combined with large NLO responses of push–pull stilbenes and their extended analogs render them suitable candidates for designing molecular NLO switches [63,64,65], which makes the current investigation of the interplay of solvation and vibrational effects on their NLO properties highly relevant also in the context of the development of reliable computational protocols for the molecular engineering of new switchable NLO materials.

## 2. Theory

In the presence of an electric field (*F*), the Cartesian component of the total dipole moment $\mu_{i}$ may be expressed as a Taylor series [66]:

(1) $$\begin{array}{cc} \mu_{i}\left(\omega_{\sigma }\right)= & \mu_{i}^{0}+\sum_{j}\alpha_{ij}\left(-\omega_{\sigma };\omega_{1}\right)F_{j}\left(\omega_{1}\right)+\\ \; & \frac{1}{2!}K^{\left(2\right)}\sum_{jk}\beta_{ijk}\left(-\omega_{\sigma };\omega_{1},\omega_{2}\right)F_{j}\left(\omega_{1}\right)F_{k}\left(\omega_{2}\right)+\\ \; & \frac{1}{3!}K^{\left(3\right)}\sum_{jkl}\gamma_{ijkl}\left(-\omega_{\sigma };\omega_{1},\omega_{2},\omega_{3}\right)F_{j}\left(\omega_{1}\right)F_{k}\left(\omega_{2}\right)F_{l}\left(\omega_{3}\right)+\ldots \; \end{array}$$

where $\mu_{i}^{0}$ is the *i*-th component of the permanent dipole moment; $\alpha_{ij}$, $\beta_{ijk}$ and $\gamma_{ijkl}$ are the components of the molecular polarizability, first and second hyperpolarizability tensors, respectively. $\omega_{\sigma }$ is the sum of frequencies $\omega_{i}$ of the interacting fields, and $K^{\left(2\right)}$ and $K^{\left(3\right)}$ are factors ensuring that all hyperpolarizabilities of the same order have the same static limit. Under the Born–Oppenheimer (BO) approximation, the molecular (hyper)polarizabilities defined by Equation (1) may be separated into pure electronic ($P^{\mathrm{el}}$) and pure vibrational ($P^{v}$) contributions, as well as the zero-point vibrational averaging (ZPVA) correction [67]:

(2) $$P=P^{\mathrm{el}}+P^{v}+P^{\mathrm{ZPVA}}\;$$

where P=α,β,γ. It is also possible to divide the property *P* into electronic, nuclear relaxation ($P^{\mathrm{nr}}$) and curvature ($P^{\mathrm{curv}}$) contributions [67]:

(3) $$P=P^{\mathrm{el}}+P^{\mathrm{nr}}+P^{\mathrm{curv}}$$

The breakdown of β into $\beta^{\mathrm{el}}$ and $\beta^{\mathrm{nr}}$ is employed in this study, and we neglect the $\beta^{\mathrm{curv}}$ contribution, as it is usually smaller than $\beta^{\mathrm{nr}}$. In order to treat the effect of molecular vibrations on electric properties, Bishop and Kirtman proposed a double (electrical and mechanical) perturbation theory (BKPT) treatment [68]. The $P^{\mathrm{nr}}$ contributions are given by the leading terms of each type of “square bracket” terms of BKPT pure vibrational contributions. Within this approach, the nuclear relaxation first hyperpolarizability may be expressed in the “square bracket” notation as (Cartesian indices are omitted for brevity):

(4) $$\beta^{\mathrm{nr}}={\left[\mu \alpha \right]}^{\left(0,0\right)}+{\left[\mu^{3}\right]}^{\left(1,0\right)}+{\left[\mu^{3}\right]}^{\left(0,1\right)}\;$$

Each square bracket involves products of the normal coordinate derivatives of the electronic electrical properties indicated, as well as harmonic vibrational frequencies and anharmonic force constants. Each term is labeled by a pair of superscripts (p,q) denoting the order in electrical and mechanical anharmonicities, respectively. In this study, the field-induced coordinates (FICs) approach is used [69,70] to determine the anharmonicity contributions to nuclear relaxation first hyperpolarizability. FICs are linear combinations of field–free normal coordinates associated with the change in equilibrium geometry induced by a static electric field.

The commonly used averaged first hyperpolarizability, $\beta_{\|}$, can be calculated from the $\beta_{ijk}$ components using the following relation [67]:

(5) $$\beta_{\|}=\frac{1}{5}\sum_{i,j}\left(\beta_{ijj}+\beta_{jij}+\beta_{jji}\right){\bar{\mu }}_{i}^{00}\;$$

In the above equations ${\bar{\mu }}_{i}^{00}$ stands for the ground-state dipole unit in the direction of the ground-state dipole moment ($\mu_{i}^{00}$/$|\mu^{00}|$).

In this study, all the mentioned quantities were computed using density functional theory (DFT) methods in combination with the PCM model (see Computational Details). To validate the applied methodology, we performed benchmark calculations and compared our results with the available experimental data. We chose to apply both these validation approaches to complement each other as neither of them can capture all aspects of the studied properties on its own. We start with a brief overview of the most relevant issues related to the comparison of theoretical and experimental data, focusing on the evaluation of the electric field-induced second harmonic generation (EFISH) signal obtained through the experimental measurements performed by Cheng et al. [39,40]. For a more in-depth discussion of the topic we point the reader to the study by Reis [71]. For any comparison to be valid, the definitions of NLO properties must be consistent. As is common for theoreticians, here we use the Taylor expansion in Equation (1), while a definition based on the power series has been applied by Cheng et al. [39,40]. Therefore, β values from Cheng’s work must be multiplied by a factor of 4 to unify our definitions. Furthermore, the vector components of β must be consistently defined as well. A numerical factor of $\frac{1}{3}$ in the expression for the EFISH signal:

(6) $$\gamma_{\mathrm{EFISH}}\left(-2\omega ;\omega ,\omega ,0\right)=\gamma_{av}\left(-2\omega ;\omega ,\omega ,0\right)+\frac{\mu^{eff}\beta^{eff}\left(-2\omega ;\omega ,\omega \right)}{3kT}$$

appears as a consequence of $\beta_{\|}$ definition in Equation (5) for the calculation of total molecular EFISH signal $\gamma_{\mathrm{EFISH}}$ within the small-field approximation applied to Boltzmann distribution. Equation (6) differs from that used by Cheng et al. [39,40], who applied the factor of $\frac{1}{5}$ in the second term. Consequently, a multiplication factor of $\frac{3}{5}$ must be used for the experimentally measured $\beta_{\|}$ values to be consistent with our computed data. We note that $\gamma_{av}$ is the average second hyperpolarizability, and the superscripts in $\mu^{eff}$, $\beta^{eff}$ and $\gamma^{eff}$ in Equation (6) denote the *effective* quantities. The importance of the *effective* property distinction is discussed in the following paragraph. Finally, it should be noted that in the experimental setup, a reference value of $\chi^{\left(2\right)}$(−2ω;ω,ω) = 0.873 pm/V for α-quartz at 1907 nm was used. However, this value has been subject to the re-evaluation with a newer accepted value of $\chi^{\left(2\right)}$(−2ω;ω,ω) = 0.555 pm/V [72] being significantly smaller. Consequently, the experimental values measured by Cheng et al. [39,40] must be adequately re-scaled.

Having resolved the basic definitions and calibration references, a full treatment of so-called local field effects is required for consistent comparisons between theory and experiments. The local field effects describe the system polarization due to the application of external electric field(s), and the way they are included in a theoretical model significantly affects computed properties. In principle, a distinction between *solute* and *effective* molecular properties can be made, the former being defined with respect to the local field acting directly on the solute molecule, while the latter refer to the response of the whole system to the applied external field. In the experimental setup, the *effective* properties are always measured. On the other hand, theoretical calculations are limited by the fact that not all solvation models allow for the full description of the medium polarization by the external field. In continuum models, it is common that only a partial system polarization is taken into the account; while the medium response to the polarization of the solute is included, the solvent is not directly polarized by the incoming field. Consequently, the properties computed in this way are neither *solute* nor *effective* and can be labeled as the *reaction-field* properties, with the term referring to the theory of cavity ($f^{C}$) and reaction field ($F^{R}$) factors as defined in the Onsager self-consistent reaction field model [73] by Wortmann et al. [74] and later corrected by Munn et al. [75]. These factors are defined as:

(7) $$f_{ij}^{C\omega }=\frac{\epsilon^{\omega }}{\epsilon^{\omega }-\kappa_{i}\left(\epsilon^{\omega }-1\right)}\delta_{ij}$$

(8) $$f_{ij}^{R\omega }=\frac{3\kappa_{i}\left(1-\kappa_{i}\right)\left(\epsilon^{\omega }-1\right)}{4\pi \epsilon_{0}a_{x}a_{y}a_{z}\left[\epsilon^{\omega }-\kappa_{i}\left(\epsilon^{\omega }-1\right)\right]}\delta_{ij}$$

(9) $$F_{ii}^{R\omega }=\frac{1}{1-f_{ii}^{R\omega }\alpha_{ii}^{sol}\left(-\omega ;\omega \right)}$$

where $\epsilon^{\omega }$ is the relative permittivity of the solvent, $\delta_{ij}$ is the Kronecker delta (*i*, *j* = *x*, *y*, *z*), $\alpha^{sol}$ is the *solute* polarizability of the solute molecule, and $a_{x}$, $a_{y}$ and $a_{z}$ are the radii parameters of the ellipsoidal cavity. The depolarization $\kappa_{i}$ is calculated as:

(10) $$\kappa_{i}=\frac{1}{2}a_{x}a_{y}a_{z}\int_{0}^{\infty }ds{\left[\left(s+a_{x}^{2}\right)\left(s+a_{y}^{2}\right)\left(s+a_{z}^{2}\right)\right]}^{-1/2}{\left(s+a_{i}^{2}\right)}^{-1}$$

where the relation $\kappa_{x}$ + $\kappa_{y}$ + $\kappa_{z}$ = 1 holds and implies $\kappa_{x}$ = $\kappa_{y}$ = $\kappa_{z}$ = 1/3 for the spherical cavity approximation. Using these factors, the relations between *solute*, *reaction-field* and *effective* properties are:

(11) $$\mu_{i}^{eff}=f_{ii}^{C0}\mu_{i}^{sol}=f_{ii}^{C0}F_{ii}^{R0}\mu_{i}^{vac}$$

(12) $$\alpha_{ii}^{eff}\left(-\omega ;\omega \right)=f_{ii}^{C\omega }\alpha_{ii}^{rf}\left(-\omega ;\omega \right)=f_{ii}^{C\omega }F_{ii}^{R\omega }\alpha_{ii}^{sol}\left(-\omega ;\omega \right)$$

(13) $$\begin{array}{ccc} \beta_{ijk}^{eff}\left(-2\omega ;\omega ,\omega \right) & = & f_{ii}^{C2\omega }f_{jj}^{C\omega }f_{kk}^{C\omega }\beta_{ijk}^{rf}\left(-2\omega ;\omega ,\omega \right)\\ & = & \left(f_{ii}^{C2\omega }F_{ii}^{R2\omega }\right)\left(f_{jj}^{C\omega }F_{jj}^{R\omega }\right)\left(f_{kk}^{C\omega }F_{kk}^{R\omega }\right)\beta_{ijk}^{sol}\left(-2\omega ;\omega ,\omega \right) \end{array}$$

where $\mu^{vac}$ is the dipole moment of the isolated molecule.

For the more advanced PCM model [76,77], a bridge between the computed *reaction-field* and *effective* properties can be gaped using the cavity field factors applying the protocol proposed by Cammi et al. [78,79]. Complementarily, the molecular solute properties can be derived from experimentally measured effective quantities using the appropriate Lorentz factors:

(14) $$f^{C\omega }F^{R\omega }=L^{\omega }=\frac{\epsilon^{\omega }+2}{3}$$

These factors are however highly approximate. To increase the accuracy, the Onsager factors (Equations (7)–(9)) were used by some authors including Cheng et al. [39] assuming a spherical cavity and isotropic polarizabilities:

(15) $$f^{C\omega }F^{R\omega }=\frac{\epsilon^{\omega }\left(\epsilon_{sol}^{\omega }+2\right)}{\left(2\epsilon^{\omega }+\epsilon_{sol}^{\omega }\right)}$$

where $\epsilon_{sol}$ is a fictitious permittivity of the solute determined from the refractive index measurements.

## 3. Results and Discussion

### 3.1. Solvent Effects on the Static Electronic Hyperpolarizability

**Figure 2:**
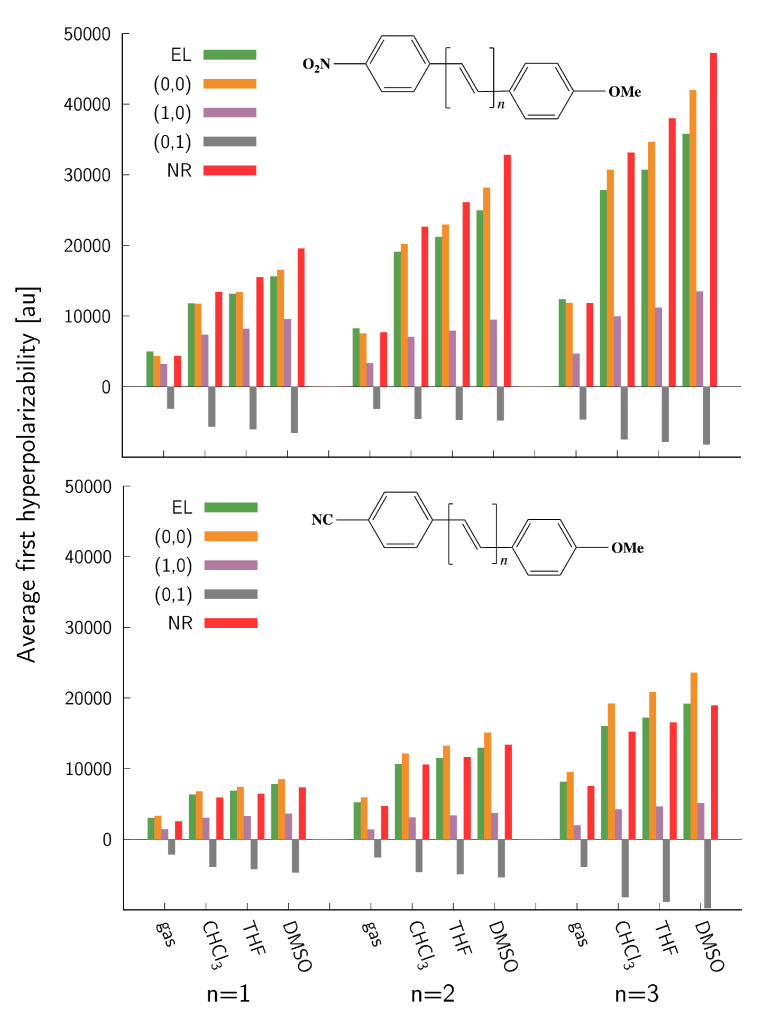
Boltzmann-weighted electronic and vibrational contributions to $\beta_{\|}$, D = OMe, A = NO${}_{2}$ (series **1**, **top**) and D = OMe, A = CN (series **2**, **bottom**).

Extended π-electron delocalization in α,ω-diphenylpolyenes involving two aromatic moieties linked via a chain of π-conjugated double bonds combined with the asymmetry introduced by strong electron donor and acceptor groups at the opposite sides results in high longitudinal electronic second-order NLO responses. All investigated molecules keep a co-planarity of the aromatic rings, and thus the π-conjugation of the whole system can be fully exploited. For short and medium-sized oligomers, the elongation of the linker usually leads to an increase in the average electronic first hyperpolarizability ($\beta^{\mathrm{el}}$) due to enhanced π-electron delocalization as also demonstrated for series **1** and **2** in Figure 2 (results for each conformer are presented in Figures S3–S18 in the Supporting Information). In general, the nitro derivatives (series **1** in Figure 2) exhibit about two-fold static $\beta^{\mathrm{el}}$ values compared to the cyano derivatives (series **2** in Figure 2), and the values increase by about a factor of 2.5 when going from *n* = 1 to *n* = 3. These observations can be rationalized using a two-state model [80], which shows that the larger second-order NLO responses of the nitro derivatives stem mainly from their larger dipole moments in the charge-transfer S${}_{1}$ state and the smaller S${}_{0}\to$ S${}_{1}$ vertical excitation energies (VEEs), while the enhancement of the response with the chain elongation arises predominantly from the decrease in VEE accompanied with the increased oscillator strength (Table S1). Conformational distortions of the methoxy group do not appear to significantly affect the electronic hyperpolarizabilities (differences between conformers **I** and **II** are typically 2–5%). On the other hand, solvent effects bring about a dramatic enhancement of the property for all investigated systems. In particular, the gas phase values are in the range of 0.5–1.3 × 10${}^{4}$ a.u. and 0.3–0.8 × 10${}^{4}$ a.u. for the series **1** and **2** in Figure 2, respectively, whereas they rise to 1.2–2.8 × 10${}^{4}$ a.u. and 0.7–1.9 × 10${}^{4}$ a.u., respectively, in chloroform (CHCl${}_{3}$) and are further elevated in more polar solvents such as tetrahydrofuran (THF) and dimethyl sulfoxide (DMSO). A similar solvent-induced amplification of $\beta^{\mathrm{el}}$ values was reported for other push–pull systems, e.g., *para*-nitro azo-enaminones [19], and was mainly attributed to a decreased HOMO-LUMO gap caused by the stabilization of the response determining charge transfer excited state in polar solvents.

### 3.2. Solvent Effects on the Static Nuclear Relaxation Hyperpolarizability

The magnitudes of the static average nuclear relaxation first hyperpolarizability ($\beta^{\mathrm{nr}}$) values are comparable to their electronic counterparts. Interestingly, despite a different nature of the contributions, they follow similar trends, i.e., they are about twice as large for the nitro derivatives compared to nitriles and also gradually increase roughly by a factor of 2.5 when going from *n* = 1 to *n* = 3 for both series of systems. The differences between conformers **I** and **II** remain relatively small for *n* = 1 and 2 but become larger for the systems with a longer linker and can reach up to 10 and 14% for the series **1** and **2**, respectively. The solvent effects on $\beta^{\mathrm{nr}}$ are huge; in fact, they are even larger than those observed for the electronic contributions. For example, the gas phase $\beta^{\mathrm{nr}}$ value for *E*-4-methoxy-4’-nitrostilbene (i.e., series **1**, *n* = 1) increases by a factor of 3.1, 3.6, and 4.5 in CHCl${}_{3}$, THF, and DMSO, respectively. With the increasing length of a π-conjugated linker, the solvent effects on $\beta^{\mathrm{nr}}$ remain huge, although the ratio with respect to the gas phase value slightly decreases (to 2.8, 3.2, and 4.0 in CHCl${}_{3}$, THF, and DMSO, respectively, for *n* = 3). Although not as dramatically as in the case of series **1**, the solvent effects also notably enhance the second-order NLO response of cyano derivatives. The gas phase $\beta^{\mathrm{nr}}$ value for *E*-4-methoxy-4’-cyanostilbene rises by a factor of 2.4, 2.6, and 3.0 in CHCl${}_{3}$, THF, and DMSO, respectively, and, similar to series **1**, the factors slightly decrease with the increasing system size. The solvent effects also change the relative weights of the electronic and vibrational contributions. Whereas the former are slightly larger (from 52 to 55%) for all investigated systems in the gas phase, the weights of the latter increase in the solvent, especially in the case of series **1**, where they prevail over the electronic counterparts.

As described in Section 2, the $\beta^{\mathrm{nr}}$ value includes both harmonic and anharmonic (up to the first order) terms. Although the harmonic contributions mimic fairly well the magnitudes as well as the trends of total $\beta^{\mathrm{nr}}$ values for both studied series of chromophores, the magnitudes of the anharmonic contributions are far from being negligible. In particular, for series **1** the harmonic values practically match the total values in the gas phase and capture as much as ca. 90% of the $\beta^{\mathrm{nr}}$ values in the solvents. This observation results from an effective cancellation of the electrical (positive) and mechanical (negative) anharmonic contributions, which can be as large as the harmonic ones (e.g., for *n* = 1 in the gas phase) and decrease to ca. 50 and 30% for larger oligomers in the gas phase and solvents, respectively. In the case of cyano derivatives, the compensation of the anharmonic terms is less pronounced, with the mechanical anharmonic contributions being larger in magnitude than their electrical counterparts. Consequently, the harmonic contributions somewhat overestimate the total $\beta^{\mathrm{nr}}$ values, with the largest overestimation observed for *n* = 3 (by ca. 35%), although the trends remain qualitatively well reproduced. The anharmonic contributions do not follow any particular trend. Whereas the relative electrical anharmonic contributions tend to show a decreasing tendency for series **2**, in the case of nitro derivatives they drop when going from *n* = 1 to *n* = 2, but remain practically the same for the two longer oligomers. As already mentioned, the mechanical anharmonicity contributions parallel the electrical counterparts for series **1**, but do not change systematically for cyano derivatives. Interestingly, the changes in solvent polarity have only a minor effect on the anharmonic contributions.

### 3.3. Validation of the Methods

As described in Computational Details, all properties discussed so far were acquired at the CAM-B3LYP/aug-cc-pVDZ/IEF-PCM level of theory. In this section, we address the appropriateness of this approach by testing the dependence of the results on selected XCFs and comparing them with the data attained by more accurate wave function methods as well as the experiment. The non-resonant electronic NLO properties of push–pull stilbenes were theoretically investigated by several groups [16,81,82,83,84,85,86]. In general, GGA and hybrid exchange-correlation functionals were found unsuitable for reliable predictions of absolute values of electronic first hyperpolarizabilities of the push–pull systems, and even the relative increase in the values when changing the benzene π-system for stilbene was largely overestimated compared to experiment by these methods, contrary to the MP2 method providing very reasonable estimates of the ratio [16,82]. On the other hand, introducing the long-range exchange correction led to a significant improvement of the results [16], which could further be upgraded by tuning the range-separation parameters [85]. The latter strategy was demonstrated to be particularly effective in the case of second hyperpolarizability [87]. Also, the application of some highly parameterized global hybrids such as M05-2X for the prediction of non-resonant NLO properties of stilbene-like push–pull NLO chromophores was advocated [86]. The two-photon absorption (2PA) of push–pull stilbenes in solution was computationally addressed by using the implicit solvation model [88,89] as well as a sequential hybrid QM/MM molecular dynamics (MD) and QM/MM response approach [90]. These works confirmed that the long-range corrected XCFs such as, e.g., CAM-B3LYP, to a large extent also remedy the difficulties of standard DFT in the description of the resonant NLO processes. Following these results, we chose CAM-B3LYP for the studies of hyperpolarizability. Moreover, the choice of this functional is further dictated by its satisfactory performance in predicting vibrational contributions to electric properties of various orders [91,92,93,94].

To start, we summarize the results of benchmarking of the electronic-structure methods in Table 1, with more detailed data presented in the Supporting Information File (Tables S2–S5). For the CAM-B3LYP, M06-2X and MN15 methods, the calculated values of vacuum dipole moment μ agree well for both studied acceptor groups independent of the bridge size. In contrast, all DFT methods give μ values 8–23% larger compared to the higher-level wave function-based SCS-MP2 method, with larger discrepancies for systems with larger bridge sizes and nitro acceptor group. Given the good agreement between SCS-MP2 and DLPNO-CCSD(T) for the smallest systems we assume that the SCS-MP2 method performs reasonably well also for the larger bridge sizes, and thus the DFT overestimation of μ appears to be systematic, which is probably related to a delocalization error of the applied methods [95]. Similar trends have been observed in the past for analogous systems by Zouaoui-Rabah et al. [96].

In the case of the $\beta_{\|}$(0;0,0) values for systems in vacuo, the applied DFT methods vary in results, with the MN15 values being significantly larger compared to those obtained with CAM-B3LYP and M06-2X. Compared to the μ values, the latter functionals predict $\beta_{\|}$(0;0,0) in a better agreement with SCS-MP2, with the deviations not exceeding 17%. Smaller errors were observed for CAM-B3LYP, staying within 15 and 6% for series **1** and **2**, respectively. This finding is in keeping with previous studies of different conjugated systems which showed that the CAM-B3LYP functional provides the $\beta_{\|}$(0;0,0) values in a fair agreement with the MP2 method [96,97,98,99]. We note that a similarly good performance for the calculation of $\beta_{\|}$(0;0,0) was reported for the M06-2X functional [96,97,98], again in agreement with the results presented here. We can therefore conclude that the CAM-B3LYP and M06-2X functionals perform reasonably well for the calculation of the $\beta_{\|}$(0;0,0) of the studied systems, at least in the gas phase.

To assess the suitability of the CAM-B3LYP/IEF-PCM(chloroform) model used in this study for the solvated systems, we move to the calculation of dynamic NLO properties and their comparison with the experimental data [39,40] (Table 2 and Table 3). Although the results presented here do not include any vibrational contributions, we note that these are expected to be relatively small when both frequencies are from optical range and thus should not significantly affect the observed trends. Furthermore, only Boltzmann weighted values are shown here, and the reader is referred to the Supporting Information (Tables S6 and S7) for the computed values of individual conformers. For the studied systems, the abundances of **I** and **II** forms are about 45% and 55%, respectively, thus the weighted values are close to simple averages. The values of calculated properties of the individual forms slightly differ, with the conformer-**II** orientation providing μβ consistently larger by about 3–7%.

As pointed out in the Section 2, the *solute*, *reaction-field*, and *effective* properties can differ significantly. For the systems studied here, the $\mu^{eff}$ values are about 7–10% larger compared to their $\mu^{sol}$ counterparts, with a slightly larger increase for systems with the nitro acceptor group. This enhancement, albeit somewhat smaller, is on par with the transition from $\mu^{vac}$ to $\mu^{sol}$ being about 14–16%. In the case of the first hyperpolarizability, the increase from $\beta_{\|}^{rf}$(−2ω;ω,ω) to $\beta_{\|}^{eff}$(−2ω;ω,ω) is about 7–13% for ω = 1907 nm, with the largest relative enhancement reached for the short-bridge systems. Here we reemphasize that all in-solution static β(0;0,0) values presented in previous sections corresponded to the *reaction-field* quantities. While directly incomparable to the experimental measurements, they can still be used well to illustrate relative trends between different systems in solution. Taking the systems with the smallest (A = CN, *n* = 1) and largest (A = NO${}_{2}$, *n* = 3) SHG responses at optical frequencies as representative examples, we observe 91% and 98% increase in their weighted $\beta_{\|}^{vac}$(−2ω;ω,ω) values (3616 au and 17102 au, respectively) when moving from *vacuum* to the *effective* quantities listed in Table 2 and Table 3. However, a dominant part of this large β enhancement is already included at the *reaction-field* level, with the increase relative to the *vacuum* being about 72% and 83%, respectively.

Comparison of the calculated values with the experimental data shows good to moderate agreement. The most reasonable results were obtained using the CAM-B3LYP and M06-2X functionals, which predict very similar μβ values overestimating the measured signals by about 7–24%. The deviations from experiment vary depending on the system size and acceptor group, but overall the best agreement is achieved for the smallest bridge size. The MN15 method gives the largest errors in all cases, which is likely due to a larger overestimation of β compared to other tested methods (Table 1). Given the difficulties associated with the calculation of the absolute signal values, a comparison of computed signal ratios for systems with the increasing bridge length and different electron acceptor groups with the experimental data appears as a suitable addition to measure the validity of applied methods. Such an analysis shows that all DFT methods give consistently a very good estimate of studied relative μβ signal values, with the CAM-B3LYP and M06-2X functionals giving the best results in most cases (Table 4). For example, the ratios of μβ(A = NO${}_{2}$) to μβ(A = CN) for systems with the same bridge lengths computed using the CAM-B3LYP functional are 2.13, 1.98, and 1.98 which can be compared to the experimental values 2.12, 1.86, and 2.20, for *n* = 1, 2, 3, respectively.

## 4. Computational Details

Geometry optimizations were performed at the CAM-B3LYP/aug-cc-pVDZ level of theory [100,101] using *VeryTight* option as implemented in the Gaussian 16 program [102] (the optimized structures are shown in Figures S1 and S2 in the Supporting Information File). Energy minima were confirmed by the evaluation of the hessian. Vibrational contributions to the static first hyperpolarizabilities were computed using in-house codes developed by Dr. J. M. Luis (University of Girona, Spain) under the equilibrium solvation regime using the IEF-PCM model (chloroform) [103].

To validate the applied methodology, three density functional theory (DFT) methods, namely CAM-B3LYP [100], M06-2X [104], and MN15 [105], as well as spin-component-scaled second-order perturbation theory (SCS-MP2) [106] and domain-based local pair natural orbital coupled cluster method with singles, doubles, and perturbative triples (DLPNO-CCSD(T)) [107,108,109] were first employed for vacuum property calculations. For the sake of consistency, all property calculations were performed using the aug-cc-pVDZ basis set and the same structures optimized at the CAM-B3LYP/aug-cc-pVDZ/IEF-PCM(chloroform) level of theory. For the DFT methods, analytical differentiation was used to obtain the first hyperpolarizabilities, whereas for SCS-MP2 and DLPNO-CCSD(T) numerical differentiation was performed. For the DLPNO-CCSD(T) calculations, the *TightPNO* settings [110] were applied, and the generalized Romberg–Rutishauser method was used to obtain stable energy derivatives [111].

To calculate the optical properties necessary for the comparison with the experimental data the CPHF algorithm was used. The *effective* properties were computed employing the approach proposed by Cammi et al. [78,79] as implemented in Gaussian 16. Other IEF-PCM settings were kept the same as in prior calculations of vibrational contributions in solution. The abundances of the two identified conformers with different orientations of the methoxy group were determined via the Boltzmann averaging procedure based on the calculated standard Gibbs energies at the CAM-B3LYP/aug-cc-pVDZ/IEF-PCM(chloroform) level. Finally, since only the *solute* properties were reported by Cheng et al. [39,40], we did the final comparison at the same level, dividing our *effective* values by appropriate Onsager factors. We note that the authors used factors given by Equation (15) using measured values not listed in the paper. Therefore, we followed the closest possible route and used the Onsager factors given by Equations (7)–(9) in the spherical cavity approximation with the default values of static and optical dielectric constants for the IEF-PCM model for the selected chloroform solvent ($\epsilon_{r}^{0}$ = 4.71, $\epsilon_{r}^{\infty }$ = 2.09). The $\alpha^{sol}$ values needed for Equation (9) were computed self-consistently from $\alpha^{rf}$ given their relation by Equation (12). Finally, recommended values for the cavity radii $a_{r}$ in the Onsager model based on molecular volume calculations as implemented in Gaussian 16 were used.

## 5. Conclusions

The solvent effects on the static first hyperpolarizability of organic molecular NLO chromophores were examined at the CAM-B3LYP/aug-cc-pVDZ/IEF-PCM level of theory focusing on the interplay between the solute–solvent interactions and the vibrational contributions to the second-order NLO responses. Two sets of experimentally known derivatives of α,ω-diphenylpolyene oligomers containing a methoxy group and nitro/cyano groups as electron donating and electron withdrawing substituents, respectively, were selected as representative push–pull NLO systems enabling to investigate the effects of an increasing π-conjugated bridge size as well as the electron acceptor strength. The appropriateness of the applied computational level was corroborated by comparing the results with more accurate wave-function based methods as well as the available experimental data. The vibrational contributions to hyperpolarizability were included via the field-induced coordinates approach enabling to assess both harmonic and anharmonic effects (up to the first order of electrical and mechanical anharmonicity).

As far as the electronic hyperpolarizability is concerned, our findings were in line with previous observations for analogous systems showing that the solvent effects can significantly alter the property in question. In our case, the $\beta^{\mathrm{el}}$ values were enhanced by a factor of 2 when going from vacuum to chloroform and further increased with the increasing solvent polarity. The changes were slightly more pronounced for the nitro derivatives. The elongation of the bridge size caused the increase of $\beta^{\mathrm{el}}$, but the amplification due to the solvent effects remained similar.

The magnitudes of the static nuclear relaxation contributions were found to be comparable to their electronic counterparts and followed similar trends. Namely, both $\beta^{\mathrm{nr}}$ and $\beta^{\mathrm{el}}$ gradually increased with the increasing bridge size, and the ratio of their magnitudes for the two sets of chromophores was preserved, with the nitro derivatives exhibiting about twice as large responses as the nitriles. The solvent effects on $\beta^{\mathrm{nr}}$ were found to be even larger than those observed for $\beta^{\mathrm{el}}$. The largest enhancements (more than four-fold(!)) were registered for the nitro derivatives in DMSO, but also in less polar environments the solvent effects appeared to be huge. The anharmonicity also played an important role in the overall trends. The magnitudes of the electrical and mechanical anharmonic contributions to $\beta^{\mathrm{nr}}$ of the studied systems were comparable to the harmonic effects in vacuum and remained significant in solvents. However, as they were of an opposite sign, they effectively canceled each other out to a large extent, and thus the total $\beta^{\mathrm{nr}}$ values remained to be well represented by the harmonic terms. Interestingly, both anharmonic contributions were found to be notably affected by the environment but their dependence on the solvent polarity was very mild. It should be underlined that these findings need not be valid for other types of systems, and further evidence is needed before drawing any generalizations.

For electronic contributions, the applied methods were validated in a two-step process. First, the CAM-B3LYP predictions of molecular $\mu^{\mathrm{el}}$ and $\beta^{\mathrm{el}}$ were compared in the vacuum to other DFT functionals, namely M06-2X and MN15, as well as SCS-MP2 and DLPNO-CCSD(T) wave-function methods. For all tested DFT methods, the $\mu^{\mathrm{el}}$ values were found to be consistently larger compared to both SCS-MP2 and DLPNO-CCSD(T). On the other hand, it was observed that both CAM-B3LYP and M06-2X were in a fair agreement with SCS-MP2 in prediction of $\beta^{\mathrm{el}}$, with MN15 significantly overestimating the property. In the second part of the validation, we focused on the in-solution properties and their comparison to the experimental data from EFISH measurements available in the literature for the chloroform solvent. Moderately good agreement of the absolute μβ values was achieved using CAM-B3LYP and M06-2X functionals, which overestimated the experimental signals by about 7–24%. Although this could be partially attributed to the observed exaggeration of $\mu^{\mathrm{el}}$ and $\beta^{\mathrm{el}}$ values by the tested DFT methods, a definitive statement cannot be made due to the overall complexity of the theory-to-experiment comparisons of the EFISH signal; however, the relative trends between different studied systems were still well reproduced by the methods applied in this paper, especially by CAM-B3LYP and M06-2X. In light of these findings, we can conclude that the CAM-B3LYP/IEF-PCM approach used throughout this study is very well suited for the evaluation of β of studied systems in solution.

## Figures and Tables

**Table 1:** Electronic contributions to total dipole moment (μ) and average first static hyperpolarizability ($\beta_{\|}$(0;0,0)) for *n* = 1, 3 with D = OMe (conformer **I**), A = NO${}_{2}$/CN. Structures were optimized at the CAM-B3LYP/aug-cc-pVDZ/IEF-PCM(chloroform) level. Properties were calculated in the vacuum and using the aug-cc-pVDZ basis set. All values are given in a.u.

	A = NO${}_{2}$ (Series 1)				A = CN (Series 2)			
	*n* = 1		*n* = 3		*n* = 1		*n* = 3	
	$\mu^{\mathrm{vac}}$	$\beta_{\|}^{\mathrm{vac}}$	$\mu^{\mathrm{vac}}$	$\beta_{\|}^{\mathrm{vac}}$	$\mu^{\mathrm{vac}}$	$\beta_{\|}^{\mathrm{vac}}$	$\mu^{\mathrm{vac}}$	$\beta_{\|}^{\mathrm{vac}}$
CAM-B3LYP	2.997	5105	3.323	12,574	2.819	2972	3.087	7979
M06-2X	2.985	4973	3.307	12,728	2.770	2988	3.032	8269
MN15	3.026	5908	3.387	15,384	2.814	3327	3.105	9418
SCS-MP2	2.597	4720	2.754	10,893	2.554	3002	2.695	7530
DLPNO-CCSD(T)	2.64 (4)			2.57 (1)				

**Table 2:** Electronic contributions to total *solute* ($\mu^{sol}$) and *effective* dipole moment ($\mu^{eff}$), average first SHG *reaction-field* ($\beta_{\|}^{rf}$(−2ω;ω,ω)) and *effective* hyperpolarizability ($\beta_{\|}^{eff}$(−2ω;ω,ω)), products $\mu \beta_{\|}$(−2ω;ω,ω) at the *effective* and *solute* levels, and comparison with the experimental data [39,40] for *n* = 1, 2, 3 with D = OMe, A = NO${}_{2}$. All values are given in au, except products μβ, which are given in 10${}^{3}$ a.u.

*n*	*Method* ${}^{a}$	$\mu^{\mathrm{sol}}$	$\beta_{\|}^{\mathrm{rf}}$	$\mu^{\mathrm{eff}}$	$\beta_{\|}^{\mathrm{eff}}$	$\mu^{\mathrm{eff}}\beta_{\|}^{\mathrm{eff}}$	$\mu^{\mathrm{sol}*}\beta_{\|}^{\mathrm{sol}*}$
1	CAM-B3LYP	3.502	12,040	3.857	13,551	52.27	11.93
	M06-2X	3.473	11,539	3.825	12,972	49.63	11.40
	MN15	3.558	14,885	3.914	16,770	65.65	14.68
	exp						10.64
2	CAM-B3LYP	3.696	20,505	4.050	22,544	91.32	19.50
	M06-2X	3.662	20,071	4.014	22,033	88.45	19.04
	MN15	3.776	26,535	4.132	29,196	120.67	25.06
	exp						15.69
3	CAM-B3LYP	3.842	31,359	4.193	33,886	142.11	29.03
	M06-2X	3.805	31,249	4.154	33,709	140.06	28.86
	MN15	3.946	42,548	4.298	46,001	197.78	38.99
	exp						26.96

^***a***^ Structures were optimized at the CAM-B3LYP/aug-cc-pVDZ/IEF-PCM(chloroform) level. Properties were calculated using the Boltzmann weighting scheme from D = OMe-**I**/**II** conformers (see Table S6) based on their respective Gibbs free energies and using the aug-cc-pVDZ basis set and IEF-PCM(chloroform). Values of $\mu^{sol*}\beta_{\|}^{sol*}$(−2ω;ω,ω) were calculated from $\mu^{eff}\beta_{\|}^{eff}$(−2ω;ω,ω) using appropriate Onsager cavity and reaction field factors (Equations (7)–(9)).

**Table 3:** Electronic contributions to total *solute* ($\mu^{sol}$) and *effective* dipole moment ($\mu^{eff}$), average first SHG *reaction-field* ($\beta_{\|}^{rf}$(−2ω;ω,ω)) and *effective* hyperpolarizability ($\beta_{\|}^{eff}$(−2ω;ω,ω)), products $\mu \beta_{\|}$(−2ω;ω,ω) at *effective* and *solute* level, and comparison with the experimental data [39,40] for *n* = 1, 2, 3 with D = OMe, A = CN. All values are given in au, except products μβ, which are given in 10${}^{3}$ a.u.

*n*	*Method* ${}^{a}$	$\mu^{\mathrm{sol}}$	$\beta_{\|}^{\mathrm{rf}}$	$\mu^{\mathrm{eff}}$	$\beta_{\|}^{\mathrm{eff}}$	$\mu^{\mathrm{eff}}\beta_{\|}^{\mathrm{eff}}$	$\mu^{\mathrm{sol}*}\beta_{\|}^{\mathrm{sol}*}$
1	CAM-B3LYP	3.287	6225	3.554	6915	24.58	5.59
	M06-2X	3.220	6247	3.483	6941	24.18	5.52
	MN15	3.294	7282	3.562	8101	28.86	6.45
	exp						5.02
2	CAM-B3LYP	3.442	11,005	3.708	11,924	44.23	9.85
	M06-2X	3.371	11,234	3.633	12,170	44.22	9.90
	MN15	3.464	13,368	3.731	14,520	54.19	11.79
	exp						8.45
3	CAM-B3LYP	3.559	17,372	3.823	18,516	70.82	14.65
	M06-2X	3.485	17,979	3.745	19,156	71.77	14.94
	MN15	3.598	22,015	3.862	23,484	90.73	18.21
	exp						12.24

^***a***^ Structures were optimized at the CAM-B3LYP/aug-cc-pVDZ/IEF-PCM(chloroform) level. Properties were calculated using the Boltzmann weighting scheme from D = OMe-**I**/**II** conformers (see Table S7) based on their respective Gibbs free energies and using aug-cc-pVDZ basis set and IEF-PCM(chloroform) model. Values of $\mu^{sol*}\beta_{\|}^{sol*}$(−2ω;ω,ω) were calculated from $\mu^{eff}\beta_{\|}^{eff}$(−2ω;ω,ω) using appropriate Onsager cavity and reaction field factors (Equations (7)–(9)).

**Table 4:** Relative (*X*/*Y*) $\mu^{sol*}\beta_{\|}^{sol*}$(−2ω;ω,ω) signals of studied systems. *X*/*Y* values denote the bridge sizes of systems in the ratio given in the individual mid-table headings.

	$\mu^{sol*}\beta_{\|}^{sol*}$(NO${}_{2}$-*nX*-OMe)/$\mu^{sol*}\beta_{\|}^{sol*}$(CN-*nY*-OMe)			
*X*/*Y*	CAM-B3LYP	M06-2X	MN15	*exp*
1/1	2.13	2.07	2.28	2.12
2/2	1.98	1.92	2.13	1.86
3/3	1.98	1.93	2.14	2.20
	$\mu^{sol*}\beta_{\|}^{sol*}$(NO${}_{2}$-*nX*-OMe)/$\mu^{sol*}\beta_{\|}^{sol*}$(NO${}_{2}$-*nY*-OMe)			
*X*/*Y*	CAM-B3LYP	M06-2X	MN15	*exp*
2/1	1.63	1.67	1.71	1.47
3/1	2.43	2.53	2.66	2.53
3/2	1.49	1.52	1.56	1.72
	$\mu^{sol*}\beta_{\|}^{sol*}$(CN-*nX*-OMe)/$\mu^{sol*}\beta_{\|}^{sol*}$(CN-*nY*-OMe)			
*X*/*Y*	CAM-B3LYP	M06-2X	MN15	*exp*
2/1	1.76	1.79	1.83	1.68
3/1	2.62	2.71	2.82	2.44
3/2	1.49	1.51	1.54	1.45

## Data Availability

Numerical data from computer simulations are available from the corresponding authors upon request.

## Supplementary Materials

The following supporting information can be downloaded at: https://www.mdpi.com/article/10.3390/molecules27248738/s1, Figures S1–S2: Geometries of molecules from series **1** and **2**; Figures S3–S18: Chain-length dependence of electronic and vibrational contributions to first hyperpolarizability; Tables S1–S7: Results of electric property calculations using a palette of quantum-chemistry methods.
