# Supplementary material for: Hyperpolarizabilities of Push–Pull Chromophores in Solution: Interplay between Electronic and Vibrational Contributions
Source: Molecules. 2022 Dec 9;27(24):8738. doi: 10.3390/molecules27248738 (PMC9783928; doi:10.3390/molecules27248738)
Supplement: Supplementary file 1 [file molecules-27-08738-s001.zip › molecules-2055769-supplementary.pdf]

Article

SUPPORTING INFORMATION
Hyperpolarizabilities of Push-Pull Chromophores in Solution: Interplay between Electronic and Vibrational Contributions

Tomáš Hrivnák[1]*, Miroslav Medveď[2,3]*, Wojciech Bartkowiak[4], Robert Zaleśny[5]*

1 Department of Molecular Simulations of Polymers, Polymer Institute, Slovak Academy of Sciences, SK-845 41 Bratislava, Slovakia
2 Department of Chemistry, Faculty of Natural Sciences, Matej Bel University, SK-97400 Banská Bystrica, Slovakia
3 Regional Centre of Advanced Technologies and Materials, Faculty of Science, Palacký University Olomouc, 783 71 Olomouc, Czech Republic
4 Department of Physical and Quantum Chemistry, Faculty of Chemistry, Wrocław University of Science and Technology, Wyb. Wyspiańskiego 27, PL–50370 Wrocław, Poland
5 Faculty of Chemistry, Wrocław University of Science and Technology, Wyb. Wyspiańskiego 27, PL–50370 Wrocław, Poland
* Correspondence: tomas.hrivnak@savba.sk (T.H.), miroslav.medved@umb.sk (M.M.), robert.zalesny@pwr.edu.pl (R.Z.)

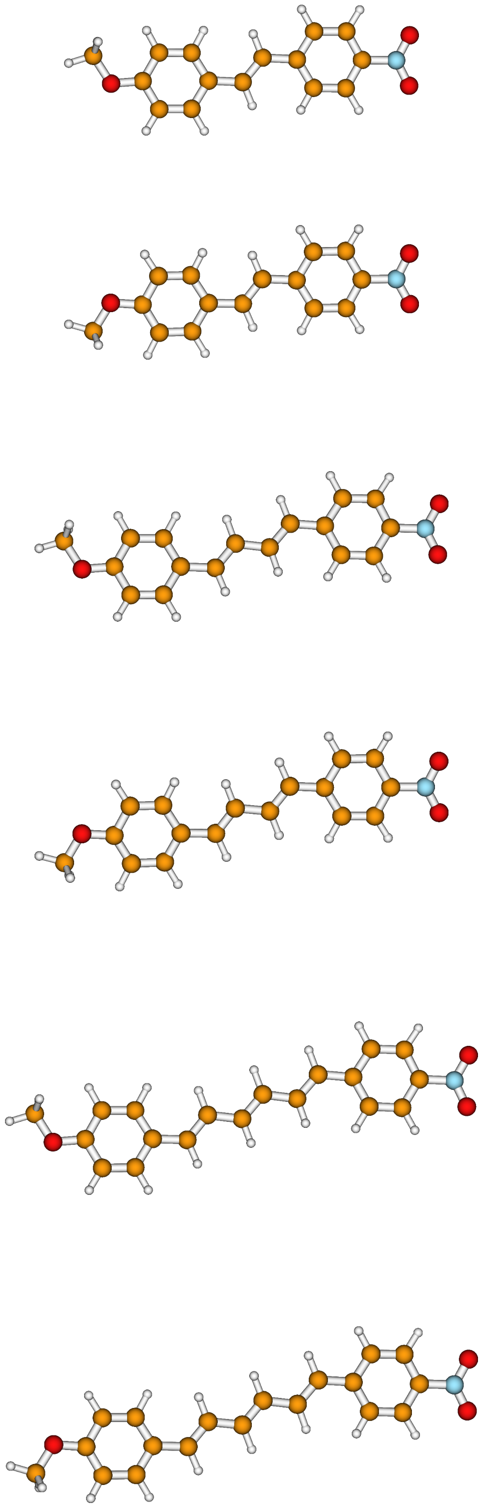

Figure S1. Geometries of molecules from series **1** optimized in the gas phase.

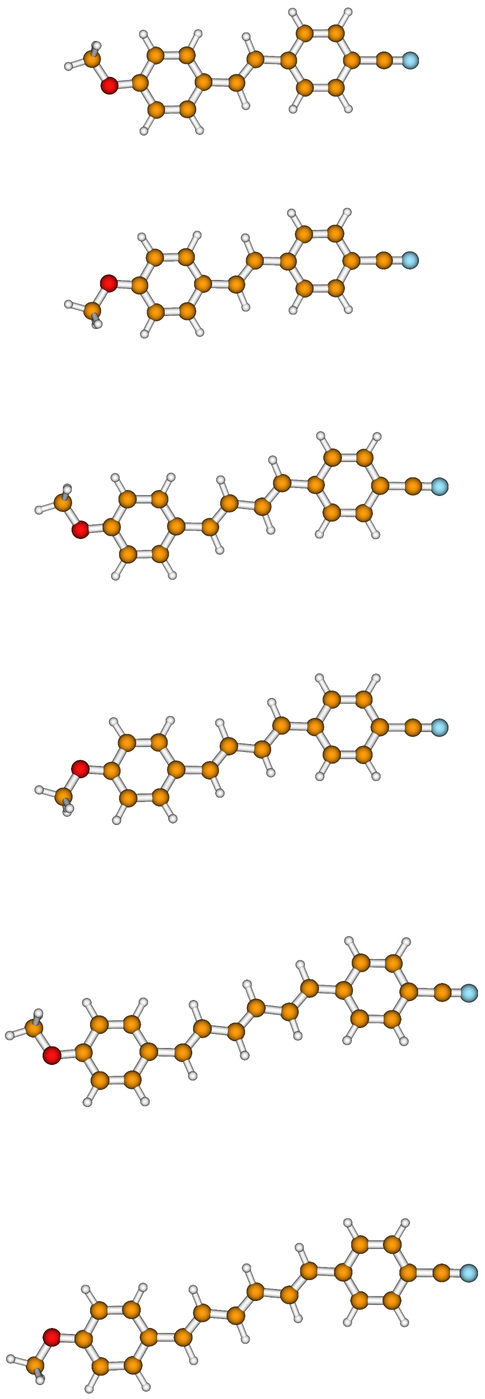

Figure S2. Geometries of molecules from series **2** optimized in the gas phase.

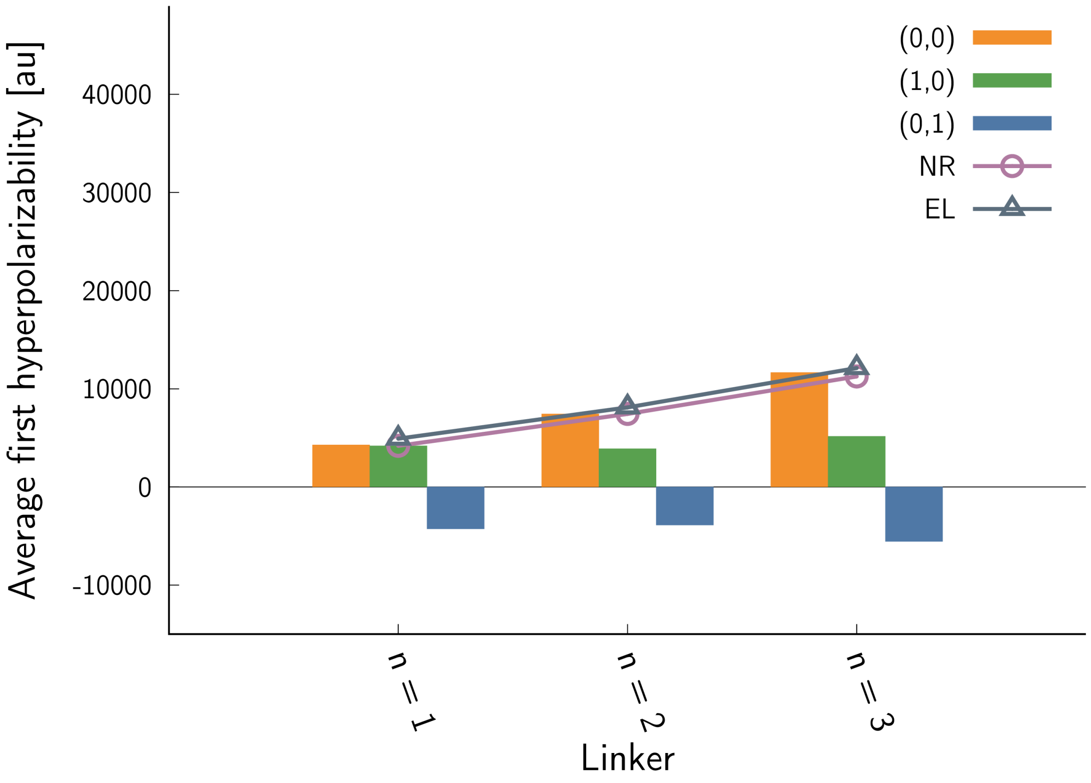

Figure S3. Chain-length dependence of electronic and vibrational contributions to β in the gas phase, D=OMe-I, A=NO_2

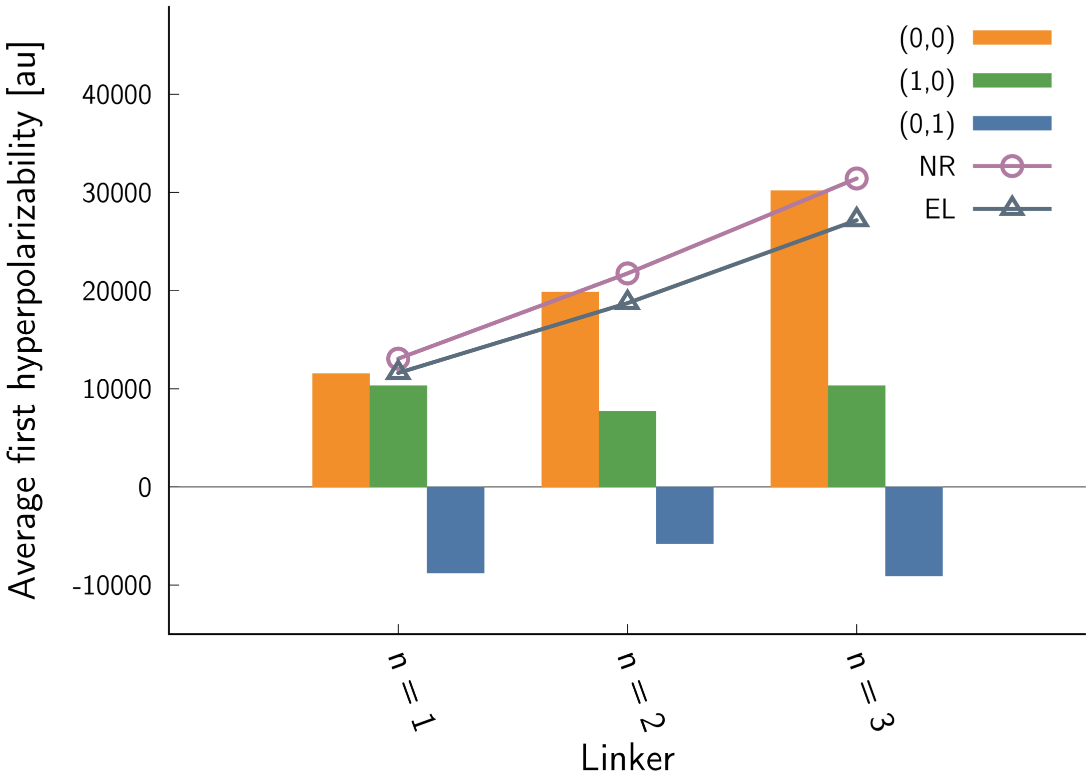

Figure S4. Chain-length dependence of electronic and vibrational contributions to β in $CHCl_3$ solution, D=OMe-I, A=NO_2

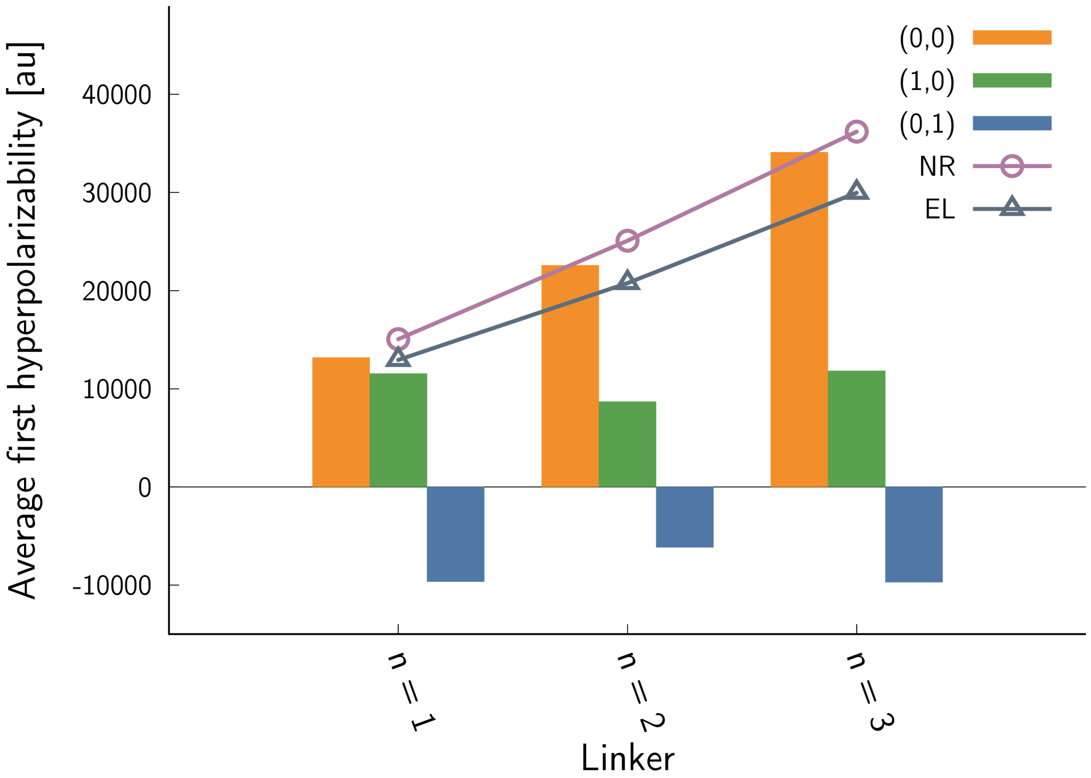

Figure S5. Chain-length dependence of electronic and vibrational contributions to β in THF solution, D=OMe-I, A=NO_2

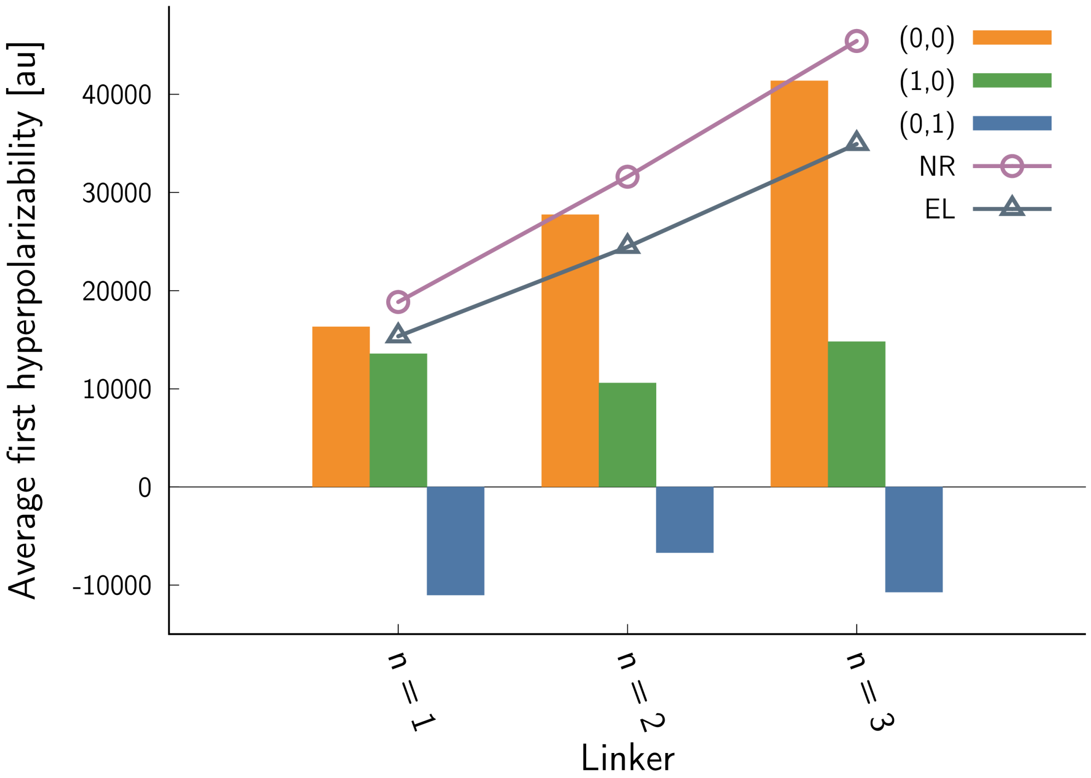

Figure S6. Chain-length dependence of electronic and vibrational contributions to β in DMSO solution, D=OMe-I, A=NO_2

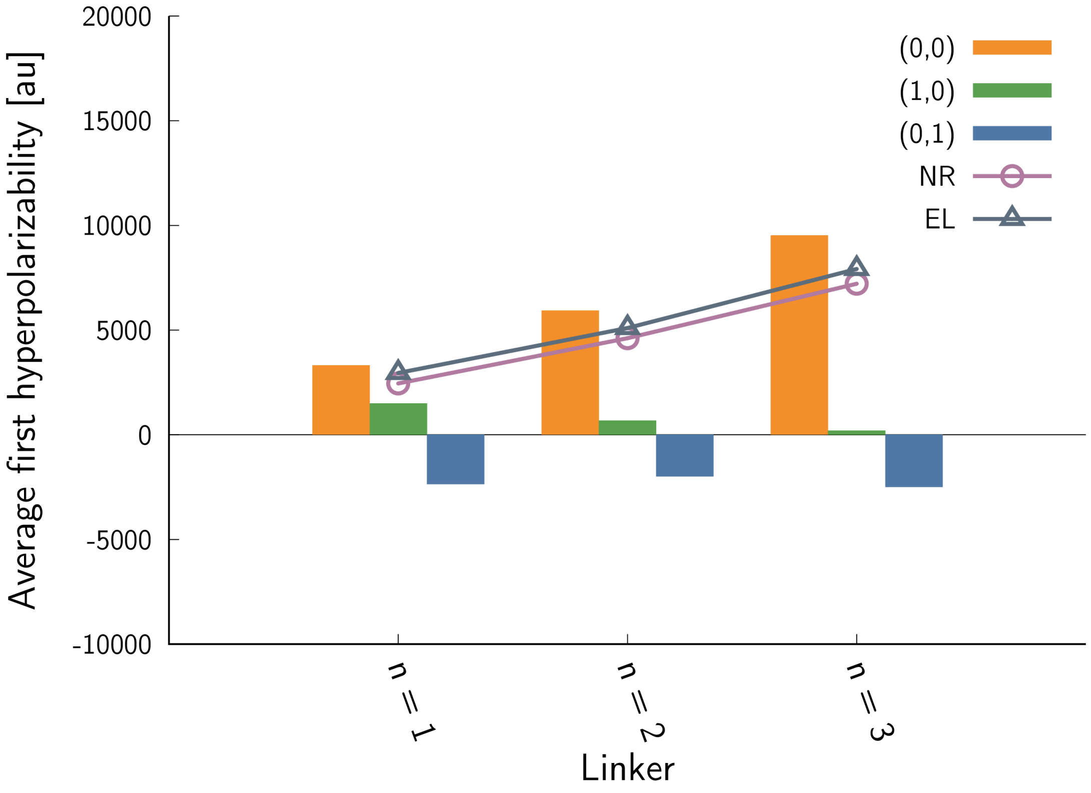

Figure S7. Chain-length dependence of electronic and vibrational contributions to β in the gas phase, D=OMe-I, A=CN

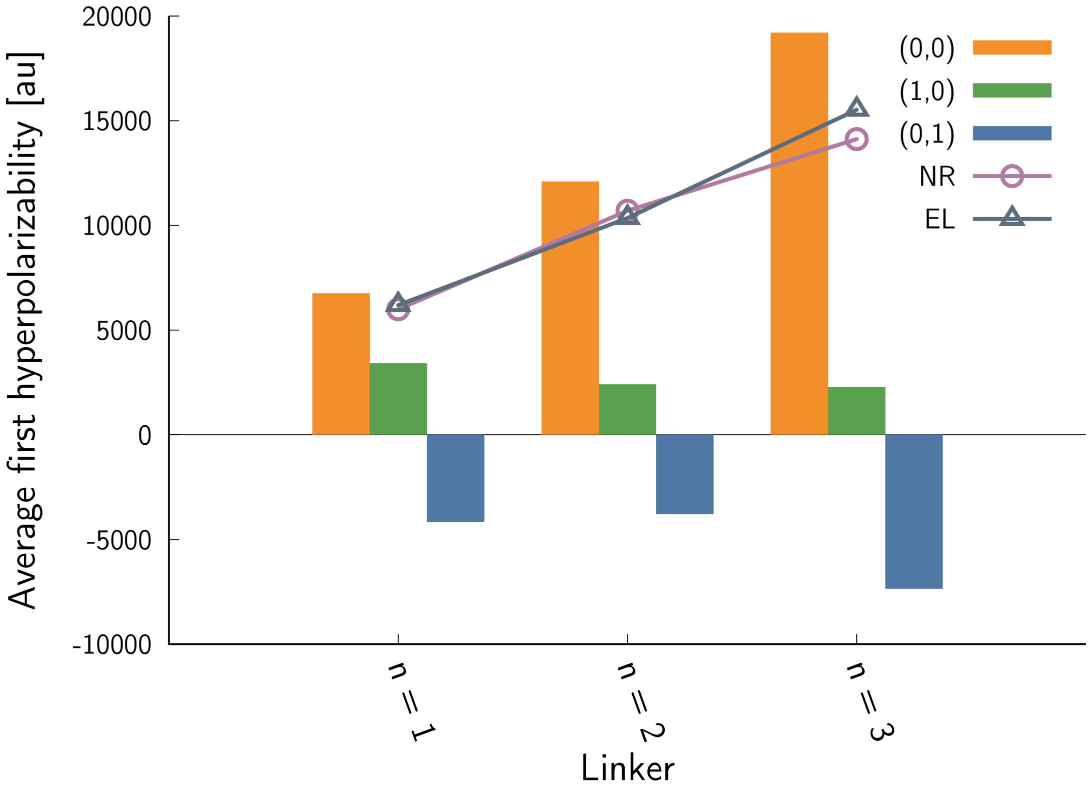

Figure S8. Chain-length dependence of electronic and vibrational contributions to β in $CHCl_3$ solution, D=OMe-I, A=CN

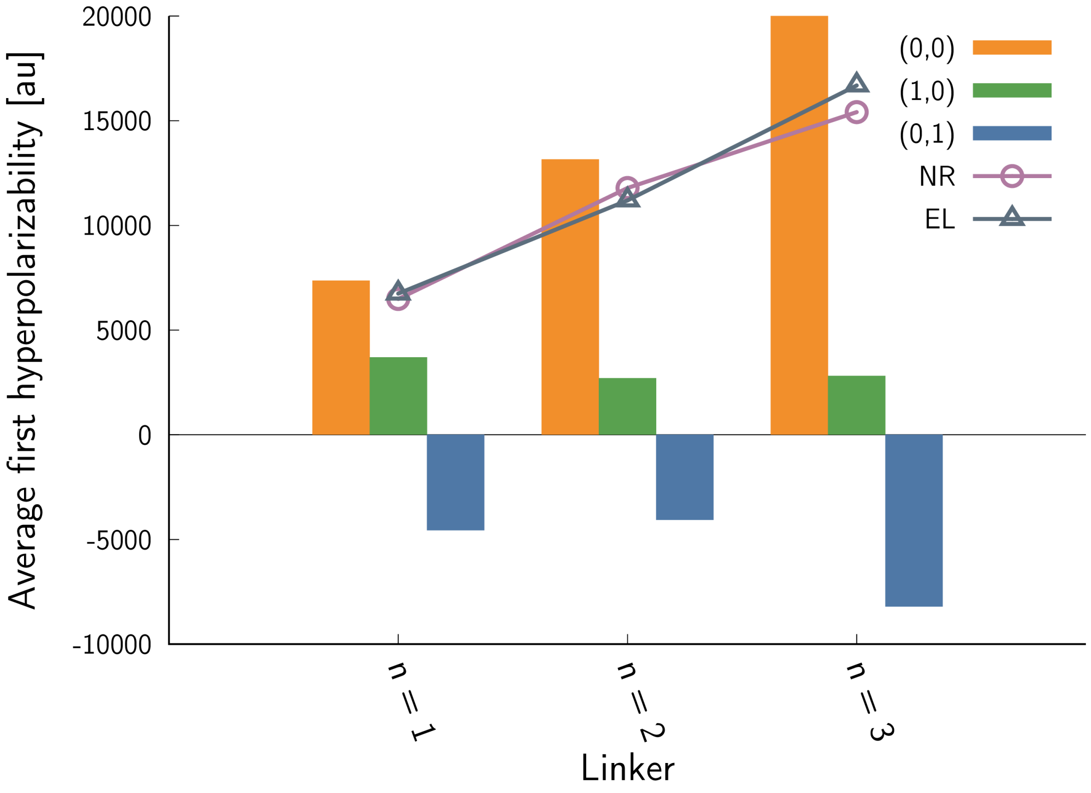

Figure S9. Chain-length dependence of electronic and vibrational contributions to β in THF solution, D=OMe-I, A=CN

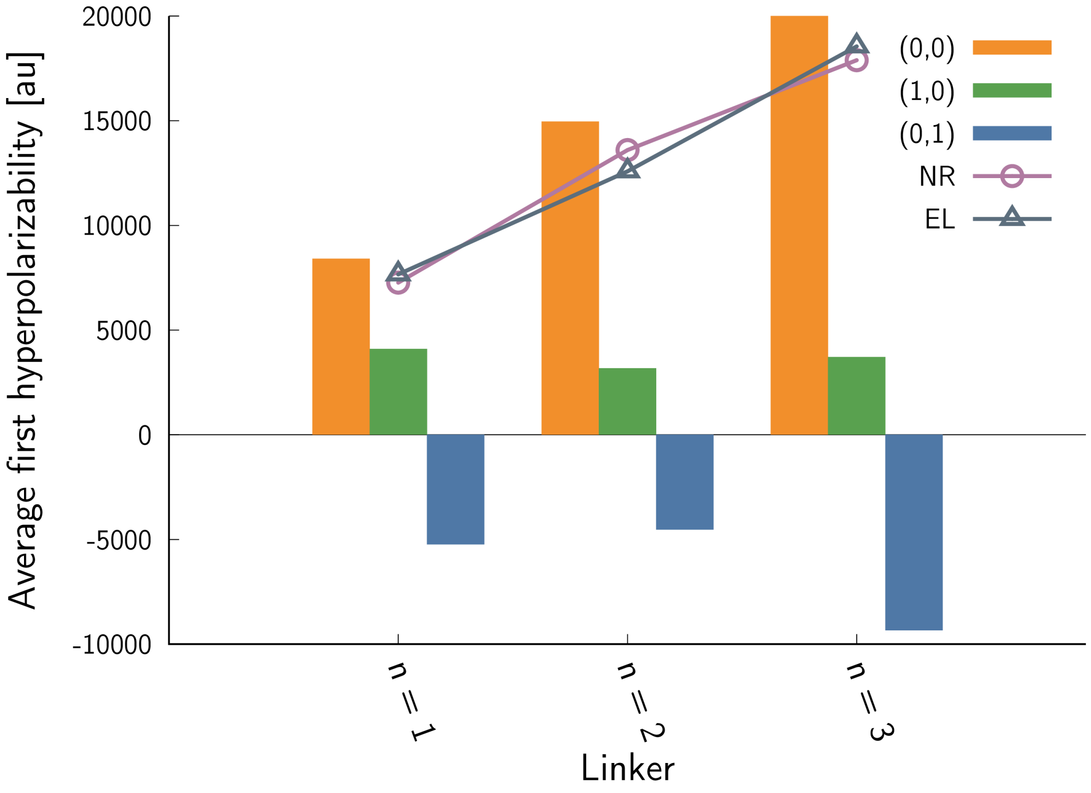

Figure S10. Chain-length dependence of electronic and vibrational contributions to β in DMSO solution, D=OMe-I, A=CN

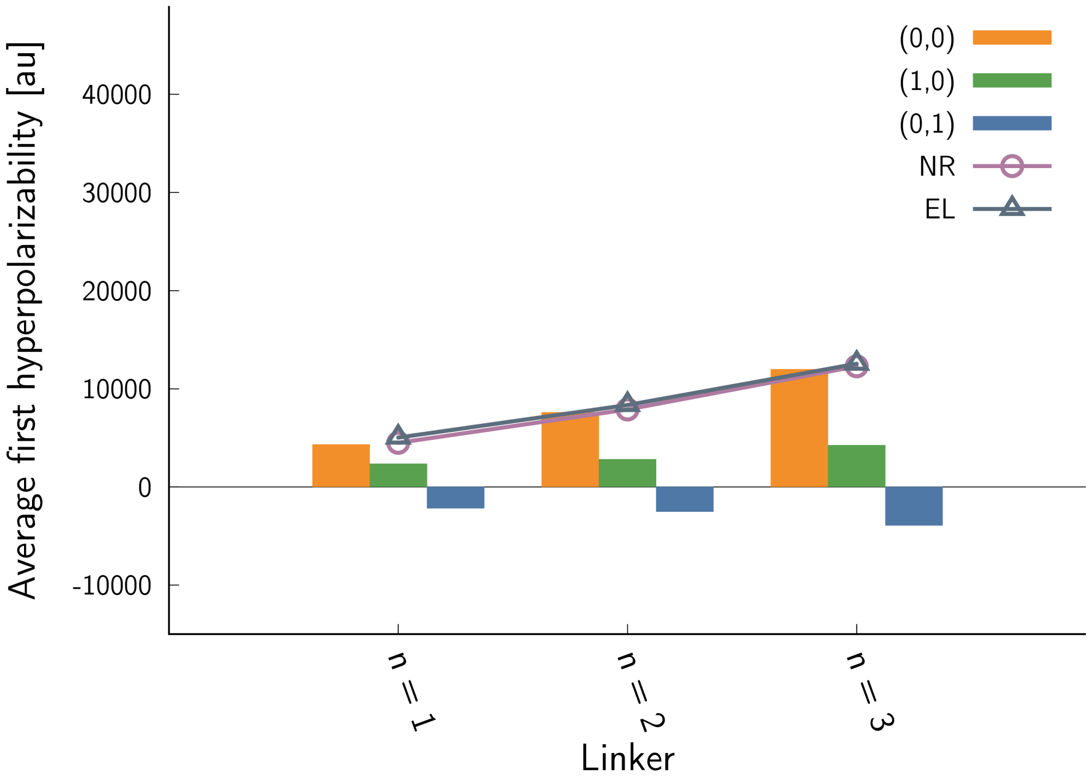

Figure S11. Chain-length dependence of electronic and vibrational contributions to β in the gas phase, D=OMe-II, A=NO_2

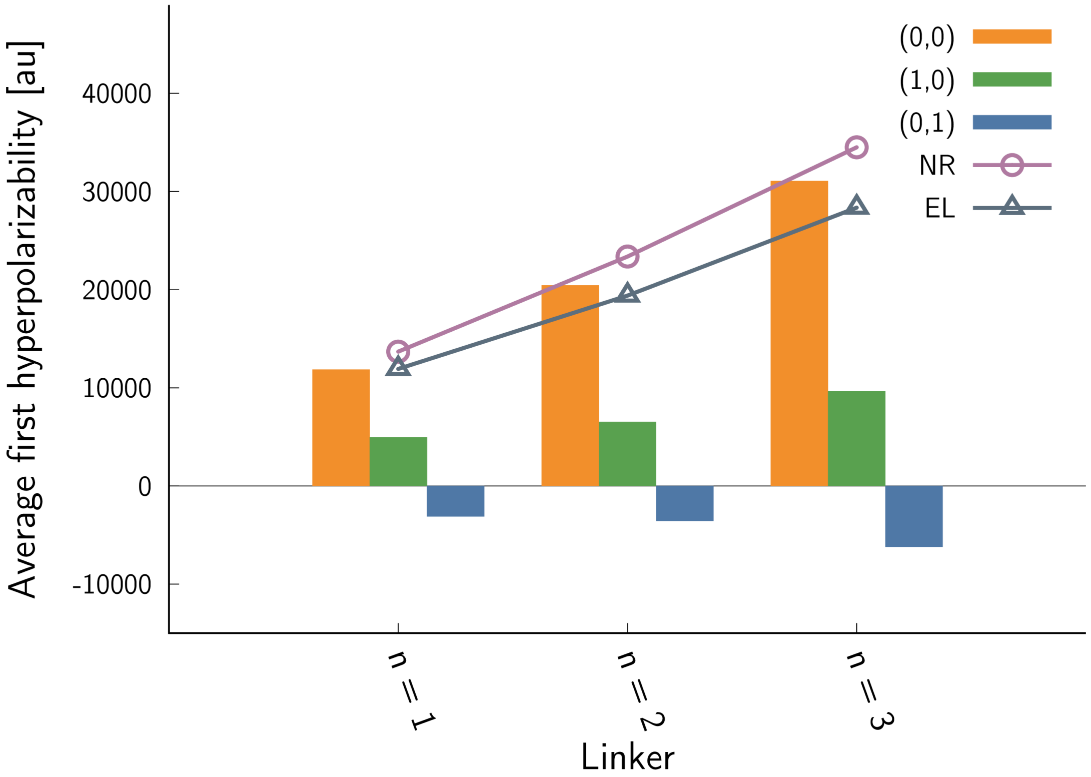

Figure S12. Chain-length dependence of electronic and vibrational contributions to β in $CHCl_3$ solution, D=OMe-II, A=NO_2

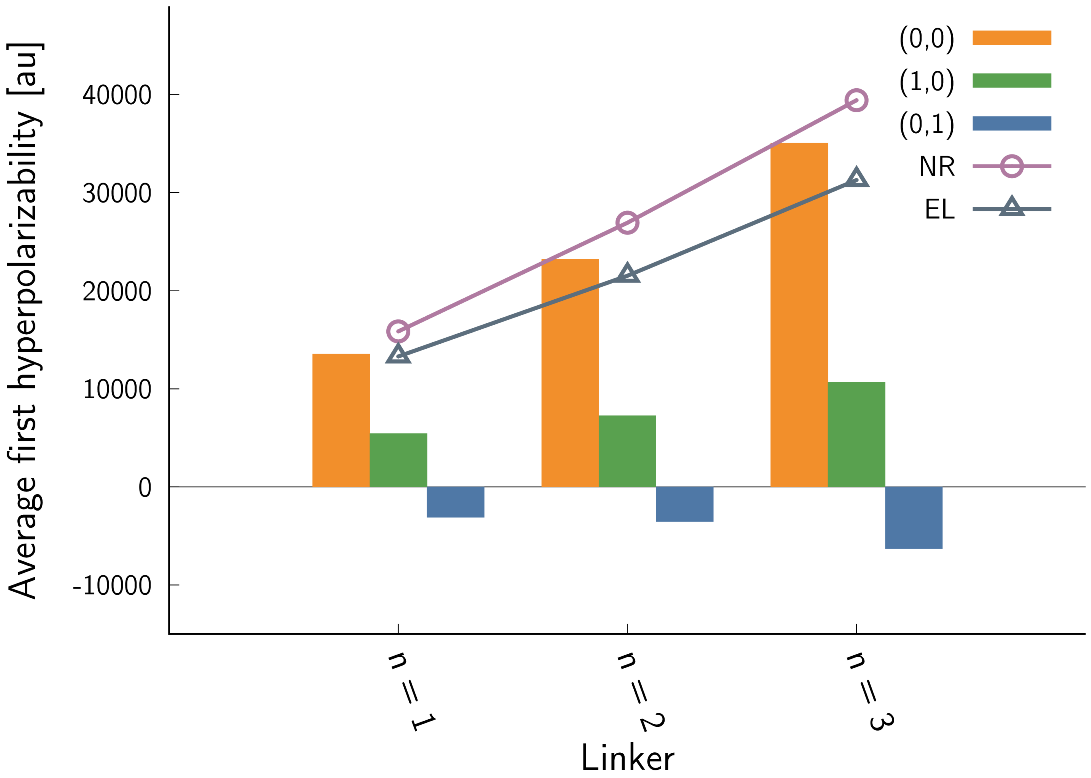

Figure S13. Chain-length dependence of electronic and vibrational contributions to β in THF solution, D=OMe-II, A=NO_2

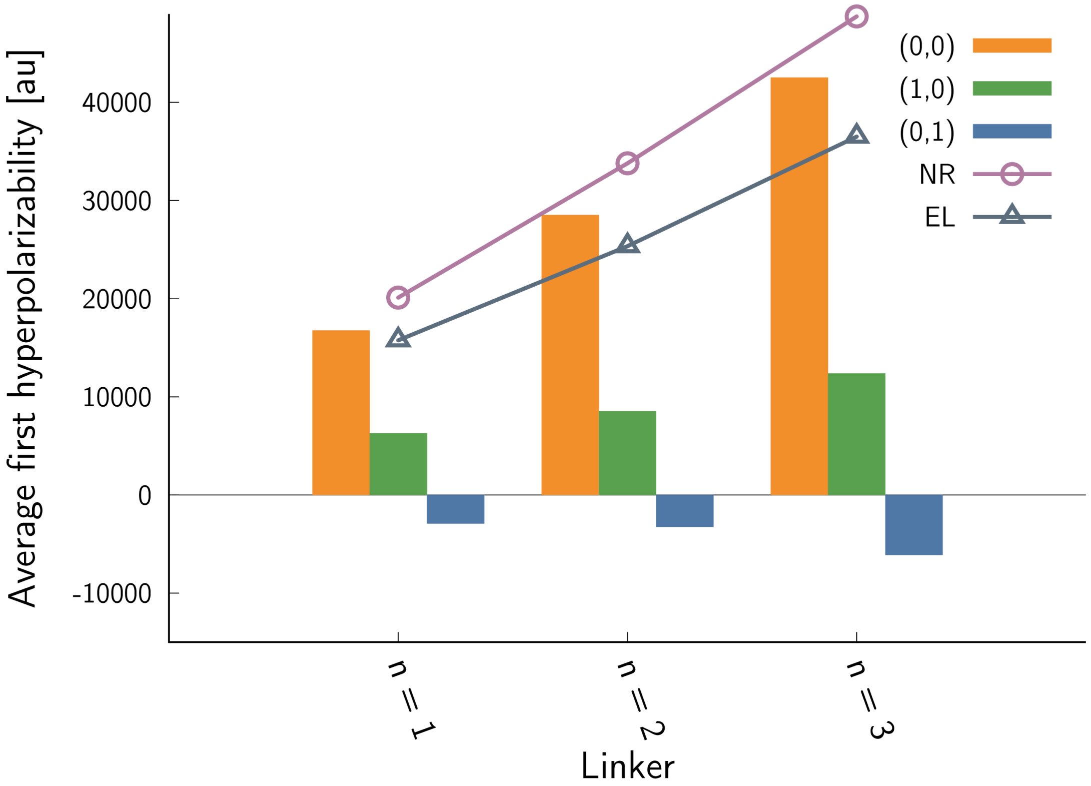

Figure S14. Chain-length dependence of electronic and vibrational contributions to β in DMSO solution, D=OMe-II, A=NO_2

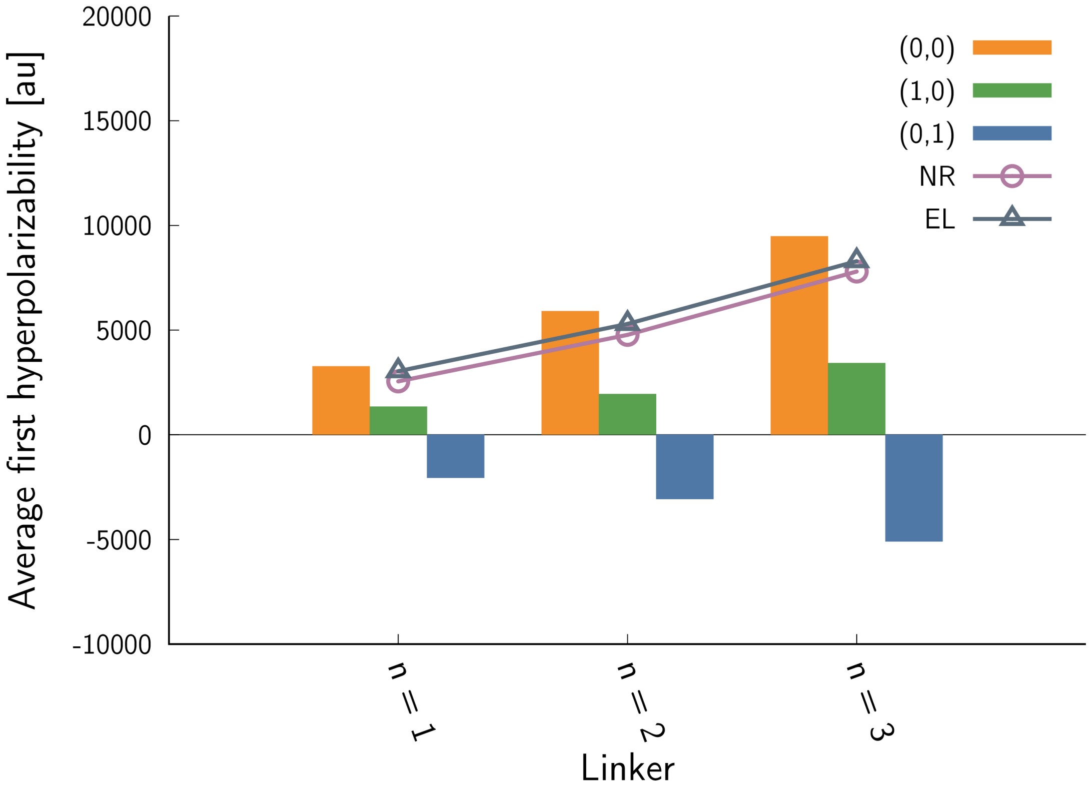

Figure S15. Chain-length dependence of electronic and vibrational contributions to β in the gas phase, D=OMe-II, A=CN

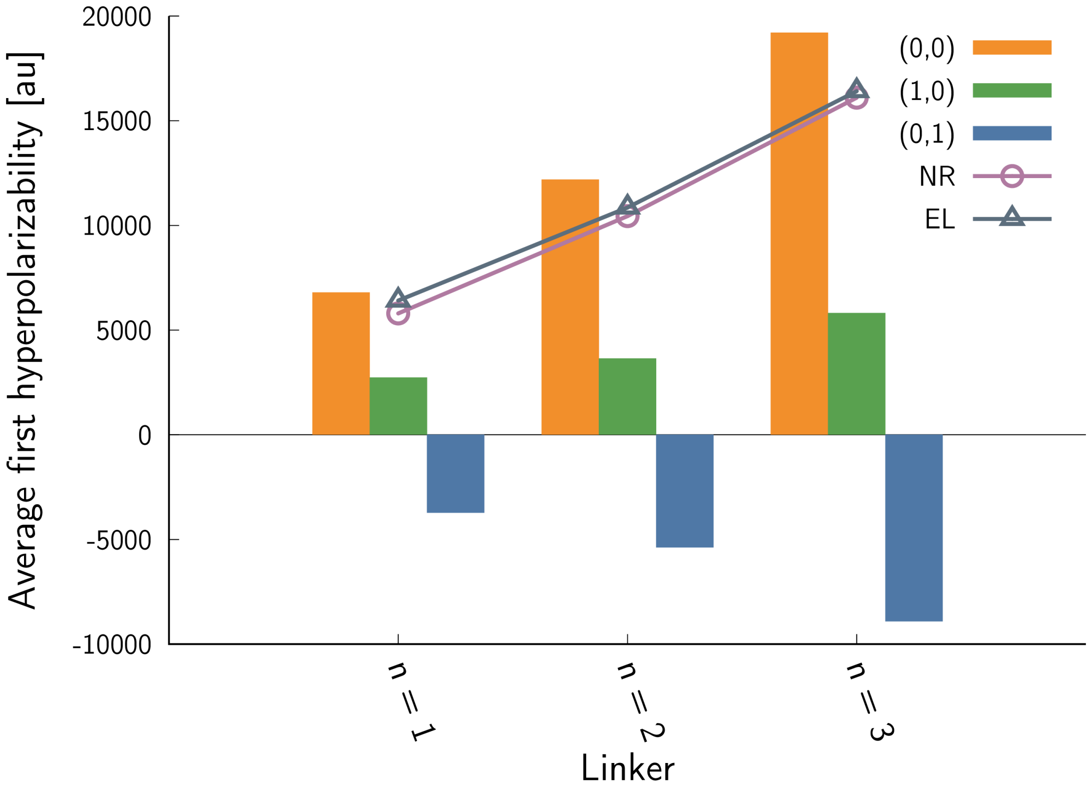

Figure S16. Chain-length dependence of electronic and vibrational contributions to β in $CHCl_3$ solution, D=OMe-II, A=CN

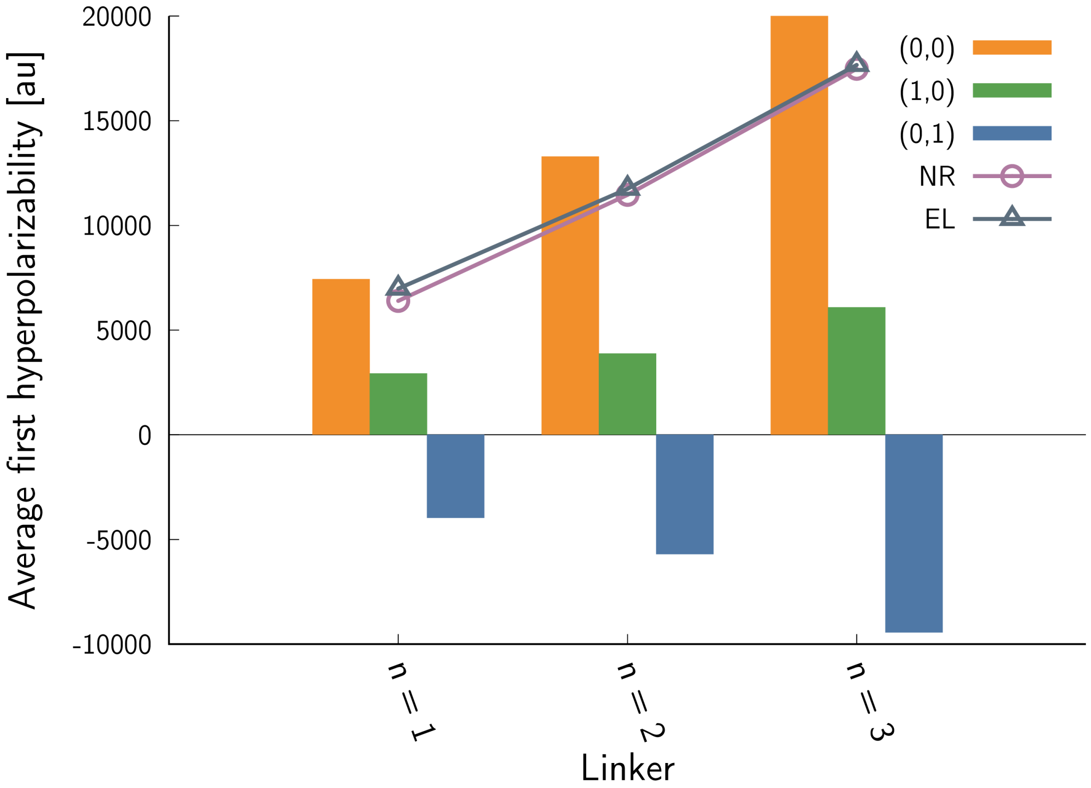

Figure S17. Chain-length dependence of electronic and vibrational contributions to β in THF solution, D=OMe-II, A=CN

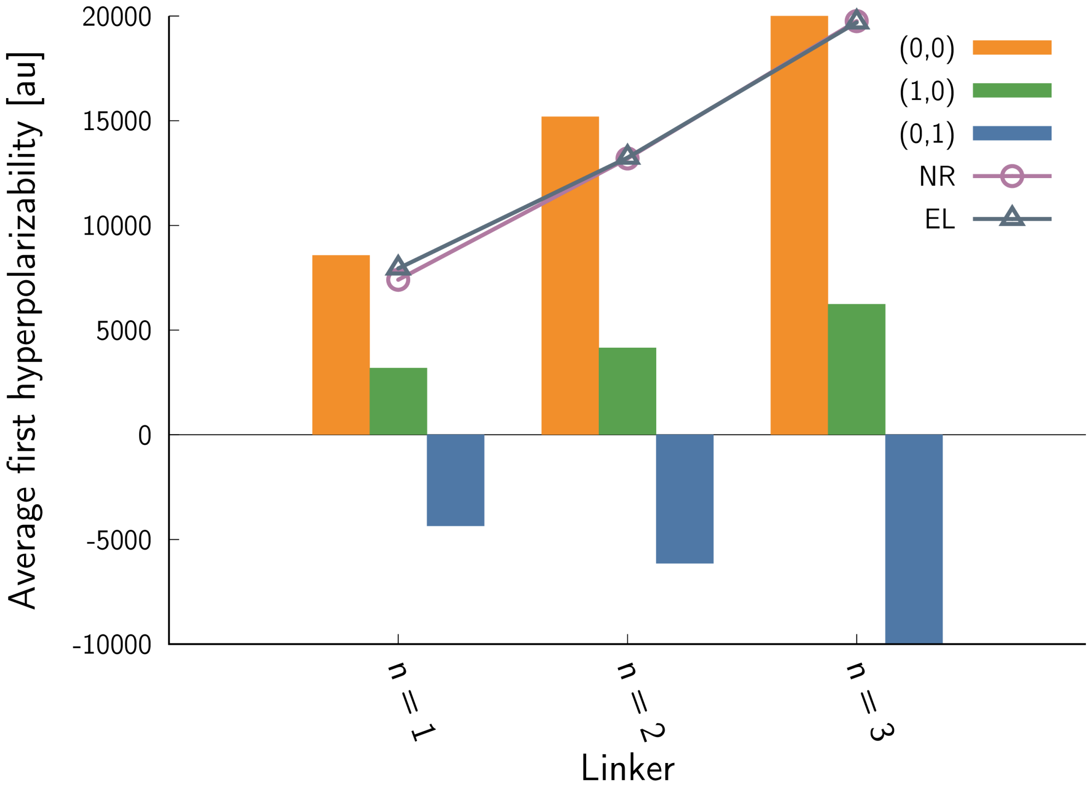

Figure S18. Chain-length dependence of electronic and vibrational contributions to β in DMSO solution, D=OMe-II, A=CN

Table S1. Estimation of the static electronic hyperpolarizability based on the two-state model (β_{2sm}) for n = 1, 2, 3 with D = OMe-I, A = NO_2/CN using the ground state (μ_g), first excited state (μ_e) and $S_0 \rightarrow S_1$ transition (μ_{ge}) dipole moments as well as the corresponding vertical excitation energies (VEEs) evaluated at the CAM-B3LYP/aug-cc-pVDZ/IEF-PCM(chloroform) level of theory. All values are given in au except for VEEs which are given in eV.

A	n	μ_g	μ_e	μ_{ge}	VEE	β_{2sm}
	1	3.48	8.55	4.07	3.28	17369
NO_2	2	3.66	9.08	4.87	3.05	30668
	3	3.79	9.34	5.61	2.87	47017
	1	3.26	6.09	4.07	3.57	8138
CN	2	3.40	6.51	4.94	3.29	15583
	3	3.51	6.72	5.70	3.06	24694

Table S2. Electronic contributions to total dipole moment (μ) and average first static hyperpolarizability ($\beta_{||}(0;0,0)$) for n = 1, 2, 3 with D = OMe-I, A = NO_2. Structures were optimized at CAM-B3LYP/aug-cc-pVDZ/IEF-PCM(chloroform) level. Properties were calculated in vacuum and using aug-cc-pVDZ basis set. All values are given in au.

	$n = 1$		$n = 2$		$n = 3$	
	μ^{vac}	$\beta_{\|\|}^{vac}$	μ^{vac}	$\beta_{\|\|}^{vac}$	μ^{vac}	$\beta_{\|\|}^{vac}$
CAM-B3LYP	2.997	5105	3.175	8407	3.323	12574
M06-2X	2.985	4973	3.161	8368	3.307	12728
MN15	3.026	5908	3.222	10001	3.387	15384
SCS-MP2	2.597	4720	2.686	7562	2.754	10893
DLPNO-CCSD(T)	2.64(4)					

Table S3. Electronic contributions to total dipole moment (μ) and average first static hyperpolarizability ($\beta_{||}(0;0,0)$) for n = 1, 2, 3 with D = OMe-II, A = NO_2. Structures were optimized at CAM-B3LYP/aug-cc-pVDZ/IEF-PCM(chloroform) level. Properties were calculated in vacuum and using aug-cc-pVDZ basis set. All values are given in au.

	$n = 1$		$n = 2$		$n = 3$	
	μ^{vac}	$\beta_{\|\|}^{vac}$	μ^{vac}	$\beta_{\|\|}^{vac}$	μ^{vac}	$\beta_{\|\|}^{vac}$
CAM-B3LYP	3.037	5212	3.231	8659	3.390	13030
M06-2X	3.023	5082	3.215	8631	3.372	13209
MN15	3.066	6020	3.278	10277	3.455	15899
SCS-MP2	2.625	4882	2.721	7953	2.792	11593
DLPNO-CCSD(T)	2.65(1)					

Table S4. Electronic contributions to total dipole moment (μ) and average first static hyperpolarizability ($\beta_{||}(0;0,0)$) for n = 1, 2, 3 with D = OMe-I, A = CN. Structures were optimized at CAM-B3LYP/aug-cc-pVDZ/IEF-PCM(chloroform) level. Properties were calculated in vacuum and using aug-cc-pVDZ basis set. All values are given in au.

	n = 1		n = 2		n = 3	
	μ^{vac}	$\beta_{\|\|}^{vac}$	μ^{vac}	$\beta_{\|\|}^{vac}$	μ^{vac}	$\beta_{\|\|}^{vac}$
CAM-B3LYP	2.819	2972	2.964	5137	3.087	7979
M06-2X	2.770	2988	2.911	5255	3.032	8269
MN15	2.814	3327	2.970	5902	3.105	9418
SCS-MP2	2.554	3002	2.633	5045	2.695	7530
DLPNO-CCSD(T)	2.57(1)					

Table S5. Electronic contributions to total dipole moment (μ) and average first static hyperpolarizability ($\beta_{||}(0;0,0)$) for n = 1, 2, 3 with D = OMe-II, A = CN. Structures were optimized at CAM-B3LYP/aug-cc-pVDZ/IEF-PCM(chloroform) level. Properties were calculated in vacuum and using aug-cc-pVDZ basis set. All values are given in au.

	$n = 1$		$n = 2$		$n = 3$	
	μ^{vac}	$\beta_{||}^{vac}$	μ^{vac}	$\beta_{||}^{vac}$	μ^{vac}	$\beta_{||}^{vac}$
CAM-B3LYP	2.858	3056	3.020	5338	3.155	8347
M06-2X	2.807	3076	2.965	5468	3.097	8663
MN15	2.852	3415	3.026	6120	3.173	9824
SCS-MP2	2.583	3124	2.671	5348	2.738	8085
DLPNO-CCSD(T)	2.56(0)					

Table S6. Electronic contributions to total *solute* (μ^{sol}) and *effective* dipole moment (μ^{eff}), average first SHG *reaction-field* ($\beta_{\|}^{rf}$(-2ω;ω,ω)) and *effective* hyperpolarizability ($\beta_{\|}^{eff}$(-2ω;ω,ω)) and products $\mu\beta_{\|}$(-2ω;ω,ω) at *effective* and *solute* level for n = 1, 2, 3 with D = OMe, A = NO_2. All values are given in au, except products $\mu\beta$, which are given in 10^3 au.

n	*method*	D = OMe-I/II	μ^{sol}	$\beta_{\|}^{rf}$	μ^{eff}	$\beta_{\|}^{eff}$	$\mu^{eff}\beta_{\|}^{eff}$	$\mu^{sol*}\beta_{\|}^{sol*}$
1	CAM-B3LYP	*I*	3.476	11871	3.822	13341	50.99	11.30
		II	3.526	12187	3.887	13735	53.39	12.48
		weighted	3.502	12040	3.857	13551	52.27	11.93
	M06-2X	*I*	3.448	11371	3.793	12761	48.40	10.80
		II	3.495	11687	3.854	13157	50.71	11.93
		weighted	3.473	11539	3.825	12972	49.63	11.40
	MN15	*I*	3.531	14697	3.879	16535	64.15	13.92
		II	3.582	15049	3.994	16976	66.96	15.35
		weighted	3.558	14885	3.914	16770	65.65	14.68
2	CAM-B3LYP	*I*	3.658	20097	4.004	22025	88.19	19.04
		II	3.727	20837	4.088	22966	93.88	19.87
		weighted	3.696	20505	4.050	22544	91.32	19.50
	M06-2X	*I*	3.626	19654	3.971	21496	85.36	18.58
		II	3.691	20411	4.049	22496	90.97	19.41
		weighted	3.662	20071	4.014	22033	88.45	19.04
	MN15	*I*	3.738	26054	4.086	28580	116.77	24.54
		II	3.808	26926	4.170	29696	123.83	25.48
		weighted	3.776	26535	4.132	29196	120.67	25.06
3	CAM-B3LYP	*I*	3.798	30601	4.141	32907	136.26	27.97
		II	3.878	31962	4.234	34664	146.77	29.87
		weighted	3.842	31359	4.193	33886	142.11	29.03
	M06-2X	*I*	3.763	30462	4.106	32686	134.20	27.79
		II	3.839	31876	4.192	34524	144.72	29.72
		weighted	3.805	31249	4.154	33709	140.06	28.86
	MN15	*I*	3.900	41609	4.246	44784	190.13	37.67
		II	3.982	43295	4.340	46968	203.86	40.04
		weighted	3.946	42548	4.298	46001	197.78	38.99

Table S7. Electronic contributions to total *solute* (μ^{sol}) and *effective* dipole moment (μ^{eff}), average first SHG *reaction-field* ($\beta_{||}^{rf}$(-2ω;ω,ω)) and *effective* hyperpolarizability ($\beta_{||}^{eff}$(-2ω;ω,ω)) and products $\mu\beta_{||}$(-2ω;ω,ω) at *effective* and *solute* level for n = 1, 2, 3 with D = OMe, A = CN. All values are given in au, except products $\mu\beta$, which are given in 10^3 au.

n	*method*	D = OMe-I/II	μ^{sol}	$\beta_{\|\|}^{rf}$	μ^{eff}	$\beta_{\|\|}^{eff}$	$\mu^{eff}\beta_{\|\|}^{eff}$	$\mu^{sol*}\beta_{\|\|}^{sol*}$
1	CAM-B3LYP	*I*	3.260	6107	3.517	6783	23.86	5.27
		II	3.308	6320	3.584	7021	25.16	5.85
		weighted	3.287	6225	3.554	6915	24.58	5.59
	M06-2X	*I*	3.194	6122	3.448	6801	23.45	5.20
		II	3.241	6346	3.511	7053	24.76	5.78
		weighted	3.220	6247	3.483	6941	24.18	5.52
	MN15	*I*	3.267	7150	3.525	7956	28.05	6.08
		II	3.316	7387	3.591	8216	29.50	6.74
		weighted	3.294	7282	3.562	8101	28.86	6.45
2	CAM-B3LYP	*I*	3.403	10702	3.659	11549	42.25	9.62
		II	3.471	11232	3.745	12206	45.71	10.02
		weighted	3.442	11005	3.708	11924	44.23	9.85
	M06-2X	*I*	3.333	10915	3.586	11770	42.21	9.66
		II	3.399	11473	3.667	12470	45.73	10.08
		weighted	3.371	11234	3.633	12170	44.22	9.90
	MN15	*I*	3.425	13039	3.682	14089	51.87	11.56
		II	3.494	13646	3.768	14843	55.93	11.97
		weighted	3.464	13386	3.731	14520	54.19	11.79
3	CAM-B3LYP	*I*	3.515	16820	3.770	17809	67.13	14.14
		II	3.594	17807	3.866	19074	73.73	15.05
		weighted	3.559	17372	3.823	18516	70.82	14.65
	M06-2X	*I*	3.443	17391	3.695	18396	67.97	14.41
		II	3.519	18442	3.785	19755	74.77	15.36
		weighted	3.485	17979	3.745	19156	71.77	14.94
	MN15	*I*	3.553	21360	3.808	22644	86.22	17.64
		II	3.633	22531	3.905	24148	94.29	18.66
		weighted	3.598	22015	3.862	23484	90.73	18.21
